# Molecular characterization of an embryonal rhabdomyosarcoma occurring in a patient with Kabuki syndrome: report and literature review in the light of tumor predisposition syndromes

**DOI:** 10.1007/s10689-022-00306-z

**Published:** 2022-07-19

**Authors:** Sietse M. Aukema, Selina Glaser, Mari F. C. M. van den Hout, Sonja Dahlum, Marinus J. Blok, Morten Hillmer, Julia Kolarova, Raf Sciot, Dina A. Schott, Reiner Siebert, Constance T. R. M. Stumpel

**Affiliations:** 1 Department of Clinical Genetics, Maastricht University Medical Centre (MUMC+), PO Box 5800, 6202 AZ Maastricht, The Netherlands; 2 Institute of Human Genetics, Ulm University and Ulm University Medical Center, Ulm, Germany; 3 Department of Pathology, Research Institute GROW, Maastricht University Medical Center, Maastricht, The Netherlands; 4 Department of Pathology, University Hospital, University of Leuven, 3000 Louvain, Belgium; 5 Department of Pediatrics, Zuyderland Medical Center, Heerlen, The Netherlands; 6 Department of Clinical Genetics and GROW-School for Oncology & Developmental Biology, Maastricht University Medical Center+, Maastricht, The Netherlands

**Keywords:** Kabuki syndrome, KMT2D, Tumor predisposition, Rhabdomyosarcoma, Methylation

## Abstract

Kabuki syndrome is a well-recognized syndrome characterized by facial dysmorphism and developmental delay/intellectual disability and in the majority of patients a germline variant in *KMT2D* is found. As somatic *KMT2D* variants can be found in 5–10% of tumors a tumor predisposition in Kabuki syndrome is discussed. So far less than 20 patients with Kabuki syndrome and a concomitant malignancy have been published. Here we report on a female patient with Kabuki syndrome and a c.2558_2559delCT germline variant in *KMT2D* who developed an embryonal rhabdomyosarcoma (ERMS) at 10 years. On tumor tissue we performed DNA-methylation profiling and exome sequencing (ES). Copy number analyses revealed aneuploidies typical for ERMS including (partial) gains of chromosomes 2, 3, 7, 8, 12, 15, and 20 and 3 focal deletions of chromosome 11p. DNA methylation profiling mapped the case to ERMS by a DNA methylation-based sarcoma classifier. Sequencing suggested gain of the wild-type *KMT2D* allele in the trisomy 12. Including our patient literature review identified 18 patients with Kabuki syndrome and a malignancy. Overall, the landscape of malignancies in patients with Kabuki syndrome was reminiscent of that of the pediatric population in general. Histopathological and molecular data were only infrequently reported and no report included next generation sequencing and/or DNA-methylation profiling. Although we found no strong arguments pointing towards KS as a tumor predisposition syndrome, based on the small numbers any relation cannot be fully excluded. Further planned studies including profiling of additional tumors and long term follow-up of KS-patients into adulthood could provide further insights.

**Supplementary Information:**

The online version contains supplementary material available at 10.1007/s10689-022-00306-z.

## Introduction

Kabuki syndrome (KS), also known as Niikawa-Kuroki syndrome or Kabuki-make-up syndrome, is a syndrome where affected persons present with a characteristic face, including arched eyebrows with a sparse lateral one-third and long palpebral fissures with eversion of the lower eyelids. Other features are hypotonia and developmental delay/intellectual disability [1]. In the majority of the patients a variant in *KMT2D* (KS1, OMIM #147920) or *KDM6A* (KS2, OMIM #300867, X-linked) can be identified [2–4]. In case of some rare syndromes, for patients, their parents and professionals involved, a key question is whether besides the syndromic features also a tumor predisposition exists [5]. This may guide tumor surveillance strategies including follow-up of the primary tumor and early detection of secondary malignancies [6]. A tumor predisposition has been established in a number of rare syndromes including Noonan syndrome type 1 (juvenile myelomonocytic leukemia), Gorlin syndrome (basal cell carcinoma, medulloblastoma) and PTEN Hamartoma Tumor (Cowden) Syndrome (predominantly breast- and thyroid carcinoma) [5, 7]. Such a predisposition is less clear for KS. Along the same lines, patients with Li-Fraumeni syndrome, Neurofibromatosis type 1, DICER1 syndrome, Costello syndrome, Noonan(-like) syndrome and Beckwith-Wiedemann syndrome are known to have an increased risk of developing rhabdomyosarcoma [8, 9]. For KS the question of a potential tumor predisposition is of special interest as somatic *KMT2D* variants are observed in approximately 5–10% of all cancers [10–13]. This frequency is even higher, up to 90%, in adult follicular lymphoma [14–18] and mutations in *KMT2D* are supposed to be driver events in various tumor types [19]. To date few (case) reports of malignancies occurring in patients with KS have been published [4, 20–35]. KMT2D fulfills as a histone 3 lysine 4 (H3K4) methyltransferase important roles in many aspects of normal development [36] and is in KS associated with a distinct DNA methylation signature [37, 38]. Also in cancer the role of KMT2D mediated DNA methylation has received increased attention [39]. Nevertheless, detailed molecular data of (epi-)genomic alterations occurring in tumors from patients with KS is lacking or sparse. Here we report on a 10-year female patient with KS who developed an embryonal rhabdomyosarcoma. On tumor material, we performed extensive molecular analyses including exome sequencing and DNA-methylation profiling. In addition we performed a review of literature focusing on the clinical-, pathological as well as molecular features of malignancies occurring in patients with KS.

## Methods

### Patient

The clinical history of the patient is described in the results section. The parents of the patient provided written informed consent for the use of archival tissue for further analyses and consent for publication.

### Histopathology and immunohistochemistry

Histopathological, immunohistochemical and FISH analyses with *FOXO1* and *EWSR1* break-apart probes were conducted as part of routine clinical diagnostics and the former re-evaluated by two bone- and soft-tissue pathologists (M.v.d.H and Ra.S.).

### Molecular and bio-informatic analyses

For the present study, after consent from the parents of the patient, additional molecular analyses were performed on fresh-frozen tumor material from the primary biopsy. For this DNA was extracted from fresh-frozen tumor tissue with a Promega Maxwell RSC DNA FFPE kit (Promega, Madison, WI, USA) according to manufacturer’s instructions.

*DNA-methylation profiling*: For DNA methylation analysis of the tumor DNA the Illumina Infinium® array technology (Illumina Inc., San Diego, CA, USA) using the Infinium Methylation EPIC BeadChip (850K array) was used following the manufacturer’s instructions. Raw methylation data was processed as analogous to Wagener et al. and further described in the Supporting methods [40, 41]. For methylation-based sarcoma classification, the DNA methylation profile of the current case was analysed using the DKFZ-sarcoma classifier (v12.2) available at https://www.molecularneuropathology.org/msp/ [42]. DNA methylation changes at the imprinted region of the Beckwith-Wiedemann-Syndrome locus at 11p15.5 in the ERMS was compared to five controls which were processed analogous to Bens et al. [43]. For copy number variant (CNV) analysis raw methylation data was normalized using the R-package minfi [44]. Subsequently, CNV data were extracted from the methylation data using the R-package conumee [45].

*Exome sequencing* was performed as described in the Supporting methods. In brief, tumor-only exome sequencing was performed on a NextSeq (Illumina, San Diego, Ca., USA) with Illumina Nextera™ Exome Kit. For data-analysis evaluation was restricted to a virtual gene panel of 95 cancer predisposition related genes (according to the TruSightCancer-Panel, Illumina) with addition of *KMT2D* and *KDM6A*, as well as 10 genes recurrently mutated in embryonal and/or fusion negative rhabdomyosarcoma (Supporting Table S1) [46–48]. The primers used for Sanger-sequencing of the germline variant in tumor material are listed in Supporting Table S2.

### Literature review

The English literature published till September 1st 2021 was searched for publications with patients with a diagnosis of KS and a concomitant malignancy. The search strategy is outlined in detail in the Supporting Methods.

## Results

### Clinical history of the patient

A 10-year old girl had a clinical and molecular diagnosis of KS with a c.2558_2559delCT pathogenic variant in *KMT2D* (g.49444907_49444908) predicted to result in a p.(Pro853Argfs*3) change at the protein level (NM_003482.3, NP_003473.3). She had the classical facial features of the syndrome as assessed by an expert dysmorphologist (C.T.R.M.S.). Her height growth followed -2 SD and she had a nasal speech. She was included in a clinical trial investigating the metabolic effect of growth hormone in children with KS [49]. However, within the first weeks of inclusion she was diagnosed with a retroperitoneal rhabdomyosarcoma and was treated with chemotherapy followed by surgical removal of the tumor. For this reason she was, according to the study protocol, excluded from the study. No causal association between the development of the rhabdomyosarcoma and initiation of growth hormone therapy was assumed.

### Histopathological and fluorescence in situ hybridization (FISH) analysis

**Fig. 1:**
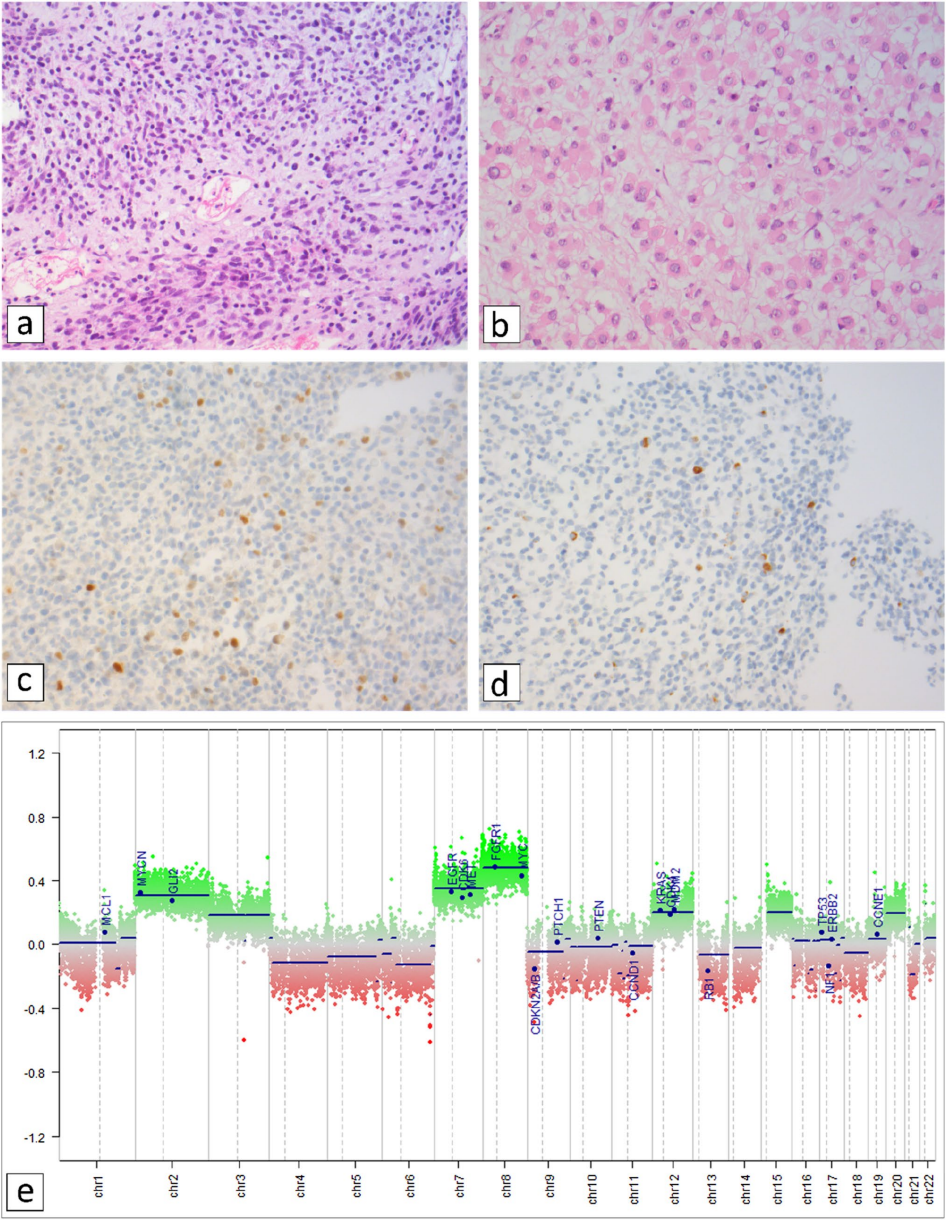
Histopathological- and molecular characterization of the tumor. **a** HE-section (X200) of the diagnostic pre-treatment core-needle biopsy showing hypercellular and less cellular areas of a small blue round cell tumor with a myxoid stromal component in the latter. **b** the excision specimen 3 months later after neoadjuvant chemotherapy was partly necrotic. Vital areas were less cellular than the primary biopsy and characterized by a more abundant myxoid matrix with tumor cells showing prominent rhabdoid differentiation as is commonly observed in embryonal rhabdomyosarcoma (ERMS) after chemotherapy [145]. In neither of the specimens an alveolar growth pattern and/or anaplastic features were seen. **c** partially positive nuclear staining for MYF4/Myogenin (**d**) and focal expression of Desmin. **e** Methylation-array based CNV profile. Gains are depicted in green, losses in red. Blue lines represent flattened profiles. Focal copy number (CN) aberrations may not be visible in the figure and genomically distinct CN aberrations laying in close proximity may not be visible as separate but instead of single genomic events. (Color figure online)

The pre-treatment core needle biopsy and post-chemotherapy excisional specimen were histopathologically analysed (Fig. 1a–d) and neither of the specimens showed an alveolar growth pattern and/or anaplastic features. With FISH analysis the tumor was *FOXO1* and *EWSR1* break negative.

### Molecular analysis

*DNA-methylation analyses*: the methylation profile obtained with genome-wide epigenomic profiling of the current case was analysed using the “DKFZ-sarcoma classifier” and showed a methylation class of (embryonal)rhabdomyosarcoma (calibration score 0.99) confirming the histopathological diagnosis. DNA-methylation based CNV-analysis showed amongst others, (partial) gains of chromosomes 2, 3, 7, 8, 12, 15, and 20 and 3 focal losses in chromosome 11p (Fig. 1e). The latter deletions at cytogenetic bands 11p15.1-p13, 11p12 and 11p11.2-q12.1 are located more centromeric than the Beckwith-Wiedemann Syndrome (BWS) locus at 11p15.5-p15.4. As patients with BWS have an increased risk of developing rhabdomyosarcomas [50] and 11p15 (epi)genetic effects are recurrent in rhabdomyosarcoma [47, 51–53], we analyzed the DNA methylation at the BWS locus in detail using the EPIC array data. Here a hypermethylation of *H19* / imprinting center 1 (IC1)/differentially methylated region 1 (DMR1) and a hypomethylation of *KCNQ1*/*KCNQ1OT1* imprinting center 2 (IC2)/differentially methylated region 2 (DMR2) was seen (Supporting Fig. S1).

*Exome sequencing* revealed, amongst others, sequence variants in *ERCC5* and *TP53.* In *KDM6A* one synonymous and in *KMT2D* one synonymous and one intronic variant were observed (Supporting Table S3). The germline variant in *KMT2D* was not identified in the tumor-DNA as a result of low-coverage at this position (exon 10, overall coverage *KMT2D* 95%) but confirmed with Sanger sequencing (Supporting Fig. S2). The Sanger sequence, moreover, suggests a gain of the wild-type and not the mutated allele in the trisomy 12 (Supporting Fig. S2, Fig. 1e). For the variants on chromosome 11 there was—in contrast to the other chromosomes—a strong bias towards variants present in a homozygous state pointing to an uniparental disomy (UPD). In contrast, the diagnostic SNP-array (trio analysis of the proband and parents) was normal (arr snp (1–22,X) × 2).

### Literature review

With literature review we identified, including the patient from the present study, 18 patients with a clinical and/or molecular diagnosis of KS who developed a malignancy (Table 1). In 10/11 patients from which DNA was subjected for mutational analysis a *KMT2D* (n = 9) or *KDM6A* (n = 1) variant was identified. One patient, patient no.8, had a clinical diagnosis of KS but was negative for *KMT2D* and *KDM6A* variants by exome sequencing and array-CGH [27]. In other reports the mutational status was not reported or were published before the identification of *MLL2* (*KMT2D*) and *KDM6A* as cause of KS in 2010 and 2012/2013 [54–56]. In eight patients without mutational status or variant (patients 2,3,7,8,10,12,13 and 14) the provided clinical features and/or photographs were compatible with a clinical diagnosis of KS. In one other patient no clinical information was provided except mentioning of the diagnosis of KS: this patient (S1) is included in Supporting Table S4. Two other patients with KS (clinical diagnosis [57] and not specified [58]) and neuroblastoma and non-Hodgkin lymphoma respectively were excluded from the literature review and discussion due to complete lack of (clinical) data of the patient, e.g. sex and age not reported. For completeness, these patients (S2 and S3) are included in Supporting Table S4.

**Table 1:** Summary of literature review of patients with Kabuki syndrome and a malignancy^a^

Pt	Sex, age	*KMT2D*/*KDM6A* variant	COSMIC/SOM	Clinical diagnosis (variant neg/NA)^b^	Malignancy^c^	Histopathological features	Molecular tumor features	Common/general (clinical) presentation for reported tumor entity?^d^	Other potential predisposing or contributing factors?^e^	Ref. case
*Bone- and soft-tissue tumors (n = 5)*										
1	F (10)	*KMT2D*, c.2558_2559delCT, p.(Pro853Argfs*3)	NO		Embryonal rhabdo-myosarcoma (ERMS, small pelvis)	Small blue round cell tumor with spindle cell features. No alveolar or anaplastic characteristics. desmin + , Myf4 ± , keratin ± , CD99 + , Myod1-, synaptophysin-	ERMS-methylation profile, typical ERMS CNVs	Typical age for ERMS. Small pelvis less common site and presentation	None^f^	*this study*
							GL-variant *KMT2D* confirmed (Het.)			
2	F (16)	NR. Normal karyotype, subtelomeric study and 22q11.2 FISH		(compatible with) Kabuki syndrome	Synovial sarcoma (SS) (right lung mass > 10 cm)	NR	NR	Common age. SS most common at extremities, trunk and head/neck. Thorax/lung is unusual but reported primary site	NR	[20]
3	F (10)	NR, clinical diagnosis. G-banded karyotype normal		(compatible with) Kabuki syndrome	Low-grade fibromyxoid sarcoma (right postero-lateral chest wall)	Myxoid background, spindle cell population, curvilinear blood vessels. Vimentin + , S100-, desmin-, SMA-, CD34-	NR	Most common in young adults, slight male preponderance. 10 yrs is typical pediatric age, Trunk common presentation	NR	[21]
4	F (10 & 12)	*KMT2D*, c.7307_7308insT (frameshift), p.(Ser2438Ilefs*11)	NO		Giant cell fibroblastoma^g^ (neck right-side)	10 yr: mesenchymal mass, vascular malformations, spindle cells, CD34 + , SMA-, S-100-, GFAP-, EMA-, Ki-67 15%	NR	Pediatric tumor. Overall male preponderance. Head- and neck region uncommon but reported. Local recurrence is common	NR	[22]
						12 yr: mesenchymal mass, vascular malformations, spindle cells, multinucleated giant cells, CD34 + , Vimentin + , SMA-, S-100-, CD99-, CD31-, EMA-, Ki-67 15%	NR			
5	F (10)	*KMT2D*, c.11233C > T, p.(Gln3745*)	YES/NO		Aggressive desmoid fibromatosis (occipital bone)	Locally invasive fibroblastic proliferation. Spindle-shaped cells, SMA +	NR	For pediatric age-group typical age. Head- and neck region less common but recurrently involved site	NR	[23]
*Hematologic malignancies (n = 5)*										
6	M (5)	*KMT2D*, c.511-1G > A, p.?	YES/YES		Burkitt lymphoma (rhino-pharyngeal mass)	“Starry-sky” pattern, CD20 + , CD79 + , CD10 + , CD38 + , BCL6 + , BCL2-, CD3-, TdT-, Ki-67 > 98%	t(8;14) + ^h^	Typical age and sex	NR	[24]
							c.511-1G > A *KMT2D* heterozygous			
7	M (3)	NR, clinical diagnosis. Normal karyotype^i^		(compatible with) Kabuki syndrome	Burkitt lymphoma (abdominal, mesenterium/ upper right quadrant, mesenteric lymphadenopathy)	Diffuse proliferation of lymphocytes. “Starry-sky” pattern, CD20 + , CD22 + , EBER-1 + (ISH), LMP-, EBNA-2-	NR	Typical age and sex	EBV +	[26]
8	M (3)	*KMT2D* and *KDM6A* negative		(compatible with) Kabuki syndrome	Burkitt lymphoma (abdominal)	NR	NR	Typical age and sex	NR/insufficient information	[27]
9	M (≥ 32)	*KMT2D*, c.12985C > T, p.(Gln4329*)	YES/YES		Hodgkin lymphoma	NR	NR	Age distribution and % EBV + vary according to (c)HL subtype	EBV + (under immunosuppression)	[28]
10	F (2)	NR; 46,XX; no 22q11 or 4p16,3 microdeletion, normal subtelomeric FISH, normal CGH		(compatible with) Kabuki syndrome	Pre-B-ALL (BM)	CD19 + , CD24 + , CD13-, CD23-, CD65S- (BM smear)	diploid (DNA index 1.00), *BCR*/*ABL*, *MLL*/*AF4*, *TEL*/*AML1*, *MLL*/*ENL* negative	Typical age for pre- B-ALL, B-ALL > > T-ALL	Maternal uncle with leukemia at age: 3 1/2 years	[29]
				CHARGE syndrome can not be fully excluded based on clinical features						
*Embryonal tumors (n = 4)*										
11	F (3)	*KMT2D*, c.13285C > T, p.(Gln4429)*	NO		Wilms tumor/nephroblastoma	Mixed-type nephroblastoma, no anaplastic features. Blastemal and epithelial elements	NR	Typical pediatric renal tumor and age	NR	[30]
12	F (0)	NR, clinical diagnosis. *PTPN11* negative, normal array-CGH		(compatible with) Kabuki syndrome	Neuroblastoma (adrenal mass)	NR	no *MYCN* amplification	Most common extracranial pediatric tumor, majority diagnosed < 5 years (median ≈18 M.)	NR	[32]
13	F (NR)	NR, clinical diagnosis		(compatible with) Kabuki syndrome	Neuroblastoma	NR	NR		NR	[31]
14	M (6)	NR, clinical diagnosis		(compatible with) Kabuki syndrome	Fetal-type hepatoblastoma	NR	NR	Most common malignant pediatric liver tumor, majority diagnosed < 5 years (median ≈ 18 M.)	NR	[32]
*Other (n = 4)*										
15	F (23)	*KMT2D*, c.16085_16086delAG, p.Lys5362Serfs*96	NO		Ependymoma (lumbar endocanalar mass, filum terminale)	Oval/elongated cells, mild nuclear atypia, eosinophilic fibrillary stroma, fascicular/ vaguely perivascular growth pattern. GFAP + , EMA + , Ki-67 3–5%	NR	Intracranial ependymoma's are more prevalent in the pediatric age group, spinal more typical presentation for adults	NR	[33]
16	F (15)	*KMT2D*, c.8594dupC	NO		Hepatocellular carcinoma (HCC)	Hepatic adenomatosis, macrovesicular steatosis, extramedullary hematopoiesis. Well-differentiated HCC with a hepatic adenoma (> 8 cm), > 10 satellite lesions	NR	OC use is associated with hepatic adenomatosis and hepatic adenomas may transform to HCC. HCC is a clinical and molecular highly heterogeneous disease	Hepatic adenomatosis, high-dose estrogen OC	[34]
17	F (16)	*KMT2D* c.2871dupA, p.(Glu958Argfs*11)	NO		Carcinoma, unknown primary origin. Lymphadenopathy from neck to abdomen, sternal, liver and kidney lesions	Cervical lymph node: poorly differentiated carcinoma, EBV-associated	NR	NA	EBV-associated?	[35]
18	F (≤ 31)^j^	*KDM6A*, c.1846dupA, p.Thr616Asnfs*5	NO		Endometrial cancer	NA	NA	≤5% of endometrial cancer diagnosed in women ≤ 40 years	NA	[4]

*B-ALL* B-cell acute lymphoblastic leukemia, *BAP* break-apart probe, *BCL *B-cell lymphoma, *BM* bone marrow, *CD* cluster of differentiation, *CGH* comparative genomic hybridization, *CHARGE syndrome/MIM#214800* coloboma, heart defects, choanal atresia, retardation of growth/development, genital- and ear abnormalities, *CNV* copy number variation, *COSMIC* catalogue of somatic mutations in cancer, *EBNA-2* Epstein-Barr nuclear-antigen 2, *EBV* Epstein-Barr virus, *EMA* epithelial membrane antigen, *FISH* fluorescence in situ hybridization, *GFAP* glial fibrillary acidic protein, *GL* germline, *Het* heterozygous, *ISH *in situ hybridization, *Ki-67* Kiel clone 67, *M* months, *Myf4* myogenin, *MYOD1* myogenic differentiation 1, *NA* not analyzed/available *Neg* negative, *NR* not reported, *OC* Oral contraceptive, *Pt* patient, *SMA* smooth muscle actin, *SOM* reported as somatic variant in COSMIC database, *TdT* terminal deoxynucleotidyl transferase, *T-ALL* T-cell acute lymphoblastic leukemia, *WHO* World Health Organization, *Yr* year

^a^Patients S1 with Burkitt lymphoma with insufficient clinical information regarding Kabuki syndrome is not present in this table but is included in Supporting Table S4

^b^Clinical diagnosis based on assessment of clinical features presented in the individual manuscripts by the author’s of the present study, see the “supplementary materials and methods” section

^c^Diagnosis, clinico- and histopathologic features as provided in the original manuscript. These may not fulfil the present WHO-criteria for the respective tumors

^d^References for common/general (clinical) presentation of individual tumor entities in Supporting data

^e^Includes (other)potential predisposing (genetic) factors for the reported malignancy

^f^Morphology does not show anaplastic features suggestive of a *TP53* germline variant [143, 144]

^g^Initial tumor diagnosed as spindle cell hemangioma, 2nd tumor at same site as giant cell fibroblastoma

^h^Diagnosed by long-distance polymerase chain reaction (PCR)

^i^Karyotype at time of tumor diagnosis (peripheral blood)

^j^Age at last examination

The reported malignancies in patients with a clinical and/or molecular diagnosis of KS are bone- and soft-tissue tumors (n = 5), hematologic malignancies (n = 5), embryonal tumors (n = 4) and tumors not belonging to any of the aforementioned malignancy groups (n = 4). Among the bone- and soft-tissue tumors, 3 of the 5 cases were reported as sarcoma (embryonal rhabdomyosarcoma, synovial sarcoma, low-grade fibromyxoid sarcoma). The most frequent hematologic malignancy was Burkitt lymphoma which was seen in 3 patients. Precursor B-cell acute lymphoblastic leukemia (pre-B-ALL) and Hodgkin’s lymphoma were seen in one patient each. Two patients presented with a neuroblastoma, and Wilms tumor, fetal-type hepatoblastoma, ependymoma, hepatocellular carcinoma, carcinoma of unknown primary origin and endometrial cancer were seen in one patient each.

In addition, the reports were screened for potential tumor predisposing and/or contributing factors other than KS. In case 10 [29], with pre-B-ALL, there was a positive family history with an uncle with leukemia at the age of 3½ years which *could* point to a familial predisposition for leukemia. Patient 16 [34], hepatocellular carcinoma (HCC), had a history of hepatic adenomatosis and use of (high-dose) oral contraceptives which could have lead or contributed to the development of HCC. Three malignancies were Epstein-Barr virus positive (EBV +): patient 7 [26] had a EBV + Burkitt lymphoma, patient 9 [28] developed an EBV + Hodgkin lymphoma under immune suppression and in patient 17 [35] an EBV-associated carcinoma of unknown primary (CUP) was diagnosed.

Although histopathological features were reported in 10 of the 16 patients (not including patient 18 with a known *KDM6A* variant) most reports lacked a detailed description. (Molecular)cytogenetic data were provided only in three published cases. For patient 6 [24], Burkitt lymphoma, it was shown that the *KMT2D* variant was present in both germ-line and tumor DNA in a heterozygous state. Apart from the case included in the present study, in none of the cases next-generation sequencing and/or methylation profiling on the tumor were performed. Although most of the tumors do not meet (current) World Health Organization (WHO) Classification of Tumours diagnostic criteria for the reported tumor entities and therefore caution has to be made with drawing conclusions based on (some of the) provided diagnoses, it appears that overall the clinical presentation of the tumors in the patients does not seem to be very unusual with regard to age and sex distribution, site of presentation and histopathological characteristics (for references, see Supporting Methods). Although patient 18 (*KDM6A* variant) developed endometrial cancer (subtype not provided) at a young age (≤ 31 years), approximately ≤5% of endometrial cancers are reported to occur in women younger than 40 years [59]. Patient 2 (synovial sarcoma) experienced a local relapse at 4 months and for patient 4 (giant cell fibroblastoma), as commonly observed for this entity [60, 61], local recurrence was reported. Further no second malignant neoplasms, bilateral-, multifocal- or meta-synchronous cancers were reported (Supporting Table S5).

## Discussion

In our study we present the history of a patient with Kabuki syndrome (KS) with a germline *KMT2D* variant who developed an embryonal rhabdomyosarcoma. On tumor-DNA we performed exome sequencing and DNA-methylation profiling and conducted a literature review focusing on the clinical-, pathological- and molecular characteristics of other malignancies occurring in patients with KS. Although patient number 18 carried a *KDM6A* variant and we cannot exclude the presence of *KDM6A* variants in the patients from the literature review in which no mutational analysis was performed or no variant was identified, a detailed discussion about the role of KDM6A in malignancies goes beyond the scope of the present study. For a detailed discussion about the (in vitro) oncogenic potential of KMT2D and its role in cancer we refer to recent articles [62–68] and reviews [10, 36, 39, 69, 70]. Also of interest in the light of tumor predisposition is the functional link between KS and RASopathies [71], a disease family with known germline predisposition to a variety of hematologic and solid cancers [72, 73]. Although based on our present analyses and literature review no definitive conclusion regarding tumor predisposition in KS can be drawn, we would like to point out several observations.

### The landscape of malignancies in patients with Kabuki syndrome

First, from a pediatric-oncology perspective it seems that the landscape of reported malignancies occurring in patients with KS broadly resembles that of the pediatric population in general. In the literature review we identified 18 patients with KS presenting with in total 19 malignancies. This included solid tumors in 13 patients (bone- and soft-tissue tumors, n = 5; embryonal tumors, n = 4; “other” tumors, n = 4) and hematologic malignancies (n = 5). The one additional patient with KS but without accompanying clinical information had a diagnosis of Burkitt lymphoma [25] (Supporting Table S4). Although having to take publication bias and the small number of cases into account this seems at least broadly in line with the different groups of malignancies occurring in the pediatric age groups in general [74]. Along the same lines, *KMT2D* would, if being a genuine tumor predisposition gene (TPG), predispose to a rather wide range of malignancies including bone- and soft-tissue tumors, hematological malignancies, embryonal tumors and carcinoma’s. Although many TPGs predispose to a single or limited types of tumors (e.g., *ATM*, 11p15/*CDKN1C*, *CDH1*, *PAX5*, *PTPN11*, *SMARCB1*), based on the observed tumor spectrum, *KMT2D* would belong to a group of TPGs predisposing to a broader spectrum of tumours like seen for *TP53*, *PTEN*, *STK11* and *DICER1*.

Of interest, although hematologic malignancies are common in both patients with KS and the general pediatric population the distribution of reported malignancies is different: for KS (including patient S1 from Table S4) 4 patients with Burkitt lymphoma [24–27] and 1 patient with B-ALL [29] have been published. In contrast, the incidence of B-ALL in the pediatric population (far) outnumbers that of Burkitt lymphoma [75, 76]. Publication bias could play a role in this, however, than one would expect this to be also the case for B-ALL and malignancies in general and not specifically for Burkitt lymphoma alone. Of interest, somatic *KMT2D* variants are recurrent but not highly frequent in Burkitt lymphoma occurring in ≤ 15% patients [77–81]. Intriguing in the light of the postulated cell-of-origin in Burkitt lymphoma—a germinal center B-cell poised to express IgA [79, 82]—and it’s pathogenesis are the reduced serum IgA levels in patients with KS and mouse models [83–85] and the smaller and reduced number of Peyer’s patches reported in one study [83]. In line with the findings of two *Kmt2d-*loss mouse models which showed (after immunization) an enhanced germinal-center response with an increase in the number of germinal-center B-cells with increased proliferation [86, 87] it might be speculated that in KS germline *KMT2D* variants may not have a direct classic tumor predisposition effect but may instead increase the chance of developing Burkitt lymphoma by an increased germinal-center response and proliferation.

As three EBV + malignancies (Hodgkin lymphoma, Burkitt lymphoma and a carcinoma of unknown primary) were reported it might be speculated that the combination of immune deficiency in patients with KS and EBV infection could—in analogy to other inborn errors errors of immunity—contribute to an increased susceptibility to develop EBV + lymphoproliferations and tumors [88, 89]. However, it has to be acknowledged that the number of EBV + malignancies in patients with KS is small and EBV-status has not been routinely reported.

## Somatic *KMT2D* variants in malignancies in patients with Kabuki syndrome and cancer in the general population

Second, (also) for other malignancies reported in patients with KS, besides Burkitt lymphoma, somatic *KMT2D* variants are mostly only infrequently reported. In contrast, malignancies in which somatic *KMT2D* variants are highly recurrent typically do not or only infrequently occur in patients with KS. E.g., somatic variants involving *KMT2D* are only infrequently reported in (embryonal) rhabdomyosarcoma [46–48, 90], in less than 10% of Hodgkin lymphoma [91–95] and on average in ≤ 15% of (pediatric) Burkitt lymphomas [77–81]. Hepatocellular carcinoma is a molecularly and clinically highly heterogeneous disease were *KMT2D* variants can be identified in approximately 5% [96–99]. In pre-B-ALL the frequency of *KMT2D* variants varies strongly between individual genetic subgroups and is high(er) in e.g. the *ERG*/*DUX4* and *ZNF384* [100, 101] rearranged subgroups but is overall, not taken these subgroups into account, low [18, 100–103]. Moreover, in the typical pediatric cancers including Wilms tumor [104–106], neuroblastoma [104, 107, 108] and pediatric hepatoblastoma [104, 109–111] somatic *KMT2D* variants only (very) infrequently occur. In contrast, in other cancers including, amongst others, pediatric- and adult diffuse large B-cell lymphoma (DLBCL) (20–35%) [11, 15, 112, 113], adult follicular lymphoma (70–90%) [14, 15], nodal marginal zone lymphoma (≈20–30%) [114–116], (non)small cell lung cancer/lung squamous cell carcinoma (≈20–30%) [11, 65, 117], upper tract urothelial carcinoma/bladder cancer (≈25–45%) [11, 118–120], esophageal (squamous cell) carcinoma (≈10–25%) [11, 121, 122] and pediatric- and adult medulloblastoma (overall ≈5–30%, large differences between individual molecular subgroups) [123–125] somatic *KMT2D* variants are (highly) recurrent but these cancers have not been reported in patients with KS (yet). However, with a lack of longitudinal studies it remains unclear whether KS patients reach the ages at which many of these tumor types are most prevalent. In cancer truncating *KMT2D* variants have been reported with varying frequencies [39, 70, 126]. A recent in-depth study analyzing germline and somatic *KMT2D* variants found 80% of the germline variants causing KS to be protein truncating. On the other hand, somatic *KMT2D* variants in cancer where predominantly missense with only 35% were predicted to be protein truncating [126]. Missense variants in patients with KS and somatic variants in cancer showed an overlapping but also different distribution across KMT2D protein domains [126]. Whereas in patients with KS many missense variants have a loss-of-function (LoF) effect (by impaired methyltransferase activity and/or loss of protein–protein interactions) [127] it can not be excluded that some missense variants in cancer may have a gain-of-function [126] or, in analogy to selected germline *KMT2D* missense variants [128] a dominant-negative effect [126]. Finally, it should be noted that the across different cancer types the percentages of missense variants varies widely [39]. Moreover, for many of the reported (presumed) somatic *KMT2D* variants no variant classification is provided [129] and depending on the cancer type may act either as (early) driver or may arise only later in the process of malignant transformation [19, 39, 130–134]. Finally, not in all studies the “true” somatic origin of the *KMT2D* variants has been reported which might be relevant considering the (relatively) frequent germline origin of *KMT2D* missense variants initially detected with sequencing of tumor material [108, 135]. Regarding germline and somatic *KMT2D* variants non-mutually exclusive parallels can be drawn with the situation for *ARID1A/B*- and *SMARCB1*. E.g. for *ARID1A/B* truncating germline variants cause Coffin-Siris-Syndrome but may not predispose to cancer although truncating somatic variants are frequently present in cancer [136, 137]. In case of *SMARCB1* both the type (truncating versus non-truncating missense) and location in the gene determine the phenotype (low-grade malignancies, malignant rhabdoid tumors, Coffin-Siris syndrome) [137]. 

### (Epi)genetic analysis of the embryonal rhabdomyosarcoma

Third, when interpreting the molecular data it should be taken into account that, in contrast to some other tumor predisposition genes, a functional read-out like bi-allelic involvement (e.g. Lynch syndrome) is not useful for *KMT2D* as overall in cancer bi-allelic variants are rare and most variants are present in heterozygous state [70]. In addition, although we did not identify a second (likely)pathogenic variant in *KMT2D* we cannot exclude such variant because of incomplete coverage (95%) of the gene. The genome wide epigenetic profiling (methylation profile, methylation changes at 11p15.5) and CNV-analysis (gains of chromosomes 2,7,8 and 12) revealed a for ERMS typical aberrations and profile [42, 47, 138]. Unfortunately, in the light of the observed trisomy 12 (Fig. 1e), we were not able to analyse the percentage of mutant versus wild-type reads at the position of the germline *KMT2D* variant due to insufficient coverage at this position and in this region. However, we confirmed the variant with Sanger sequencing. Although taking the intrinsic limitations of quantification and Sanger sequencing into account the pattern of the peak-heights of the wild-type and mutated-sequence is suggestive for a gain of the wild-type and not the mutated allele.

## Conclusions

In conclusion we present the first exome wide genomic and genome wide epigenomic analyses of a malignancy occurring in a patient with KS. Our molecular findings and observations from the literature neither prove nor rule out a potential tumor predisposition for KS. Regarding the tumor spectrum and age of onset of the tumor in patients with Kabuki syndrome it was observed that this broadly resembled that of the pediatric population in general. However, even an in vitro or in vivo oncogenic effect of KMT2D perturbation might not directly translate to a clinical relevant tumor predisposition. Regarding the latter, one should consider that if in KS the cancer-frequency would exceed a reasonable threshold for tumor surveillance (e.g. ≥ 1 ~ 5% for other pediatric cancer predisposition syndromes [139]) and if the (dis)advantages of imaging modalities like whole-body MRI (e.g. false-positive findings, required general anesthesia) outweigh the benefits [140, 141] for pediatric patients especially when they suffer from developmental delay. Moreover, considering that Burkitt lymphoma appears rather frequent in patients with KS and has a doubling time of approximately 24 h only long-term interval surveillance would not be effective. Alternatively, liquid-biopsy-based surveillance strategies might overcome some of these hurdles in paediatric patients with a cancer predisposition syndrome [142]. The (epi-)genetic analysis revealed a typical ERMS methylation- and copy number profile. Although we found no strong arguments pointing towards KS as a tumor predisposition syndrome, based on the small numbers any relation cannot be fully excluded. Further planned studies including exome- and genome-wide (epi)genetic profiling of additional tumors in patients with KS and long term follow-up of patients with KS into adulthood could provide further insights into the pathogenesis of these rare but challenging tumors.

## Supplementary Information

Below is the link to the electronic supplementary material.

Supplementary file1 (PDF 502 kb)

## Data Availability

Data available in article Supporting Information.

## References

[1] Adam MP, Banka S, Bjornsson HT (2019). Kabuki syndrome: international consensus diagnostic criteria. J Med Genet.

[2] Miyake N, Koshimizu E, Okamoto N (2013). MLL2 and KDM6A mutations in patients with Kabuki syndrome. Am J Med Genet A.

[3] Bögershausen N, Gatinois V, Riehmer V (2016). Mutation update for Kabuki syndrome genes KMT2D and KDM6A and further delineation of X-linked Kabuki syndrome subtype 2. Hum Mutat.

[4] Faundes V, Goh S, Akilapa R (2021). Clinical delineation, sex differences, and genotype-phenotype correlation in pathogenic KDM6A variants causing X-linked Kabuki syndrome type 2. Genet Med.

[5] Postema FAM, Oosterwijk JC, Hennekam RC (2020). Genetic control of tumor development in malformation syndromes. Am J Med Genet A.

[6] Kuhlen M, Wieczorek D, Siebert R, Frühwald MC (2019). How I approach hereditary cancer predisposition in a child with cancer. Pediatr Blood Cancer.

[7] Ripperger T, Bielack SS, Borkhardt A (2017). Childhood cancer predisposition syndromes-a concise review and recommendations by the Cancer Predisposition Working Group of the Society for Pediatric Oncology and Hematology. Am J Med Genet A.

[8] Skapek SX, Ferrari A, Gupta AA (2019). Rhabdomyosarcoma. Nat Rev Dis Prim.

[9] Li H, Sisoudiya SD, Martin-Giacalone BA (2020). Germline cancer-predisposition variants in pediatric rhabdomyosarcoma: a report from the children’s oncology group. J Natl Cancer Inst.

[10] Fagan RJ, Dingwall AK (2019). COMPASS ascending: emerging clues regarding the roles of MLL3/KMT2C and MLL2/KMT2D proteins in cancer. Cancer Lett.

[11] Bailey MH, Tokheim C, Porta-Pardo E (2018). Comprehensive characterization of cancer driver genes and mutations. Cell.

[12] ICGC/TCGA Pan-Cancer Analysis of Whole Genomes Consortium (2020) Pan-cancer analysis of whole genomes. Nature 578:82–93. 10.1038/s41586-020-1969-6 10.1038/s41586-020-1969-6 PMC7025898 32025007

[13] Mendiratta G, Ke E, Aziz M (2021). Cancer gene mutation frequencies for the U.S. population. Nat Commun.

[14] Huet S, Sujobert P, Salles G (2018). From genetics to the clinic: a translational perspective on follicular lymphoma. Nat Rev Cancer.

[15] Green MR (2018). Chromatin modifying gene mutations in follicular lymphoma. Blood.

[16] Hübschmann D, Kleinheinz K, Wagener R (2021). Mutational mechanisms shaping the coding and noncoding genome of germinal center derived B-cell lymphomas. Leukemia.

[17] Loeffler M, Kreuz M, Haake A (2015). Genomic and epigenomic co-evolution in follicular lymphomas. Leukemia.

[18] Stengel A, Baer C, Walter W (2021). Mutational patterns and their correlation to CHIP-related mutations and age in hematological malignancies. Blood Adv.

[19] Martínez-Jiménez F, Muiños F, Sentís I (2020). A compendium of mutational cancer driver genes. Nat Rev Cancer.

[20] Casanova M, Selicorni A, Ferrari A (2011). Cancer predisposition in children with Kabuki syndrome. Am J Med Genet A.

[21] Shahdadpuri R, O’Meara A, O’Sullivan M, Reardon W (2008). Low-grade fibromyxoid sarcoma: yet another malignancy associated with Kabuki syndrome. Clin Dysmorphol.

[22] Karagianni P, Lambropoulos V, Stergidou D (2016). Recurrent giant cell fibroblastoma: malignancy predisposition in Kabuki syndrome revisited. Am J Med Genet A.

[23] Scala M, Morana G, Sementa AR (2019). Aggressive desmoid fibromatosis in Kabuki syndrome: expanding the tumor spectrum. Pediatr Blood Cancer.

[24] de Billy E, Strocchio L, Cacchione A (2019). Burkitt lymphoma in a patient with Kabuki syndrome carrying a novel KMT2D mutation. Am J Med Genet A.

[25] Attarbaschi A, Carraro E, Abla O (2016). Non-Hodgkin lymphoma and pre-existing conditions: spectrum, clinical characteristics and outcome in 213 children and adolescents. Haematologica.

[26] Ijichi O, Kawakami K, Matsuda Y (1996). A case of Kabuki make-up syndrome with EBV+Burkitt’s lymphoma. Acta Paediatr Jpn.

[27] Cheon CK, Sohn YB, Ko JM (2014). Identification of KMT2D and KDM6A mutations by exome sequencing in Korean patients with Kabuki syndrome. J Hum Genet.

[28] Kaiwar C, Kruisselbrink TM, Kudva YC (2019). Exome sequencing confirms diagnosis of kabuki syndrome in an-adult with hodgkin lymphoma and unusually severe multisystem phenotype. Clin Immunol.

[29] Scherer S, Theile U, Beyer V (2003). Patient with Kabuki syndrome and acute leukemia. Am J Med Genet A.

[30] Teranishi H, Koga Y, Nakashima K (2018). Cancer management in kabuki syndrome: the first case of wilms tumor and a literature review. J Pediatr Hematol Oncol.

[31] Merks JHM, Caron HN, Hennekam RCM (2005). High incidence of malformation syndromes in a series of 1,073 children with cancer. Am J Med Genet A.

[32] Tumino M, Licciardello M, Sorge G (2010). Kabuki syndrome and cancer in two patients. Am J Med Genet A.

[33] Roma D, Palma P, Capolino R (2015). Spinal ependymoma in a patient with Kabuki syndrome: a case report. BMC Med Genet.

[34] Timothy LD, Lehrke HD, Chandan VS (2019). Diffuse adenomatosis and hepatocellular carcinoma treated with liver transplantation in an adolescent female with kabuki syndrome with a novel KMT2D gene mutation. Case Rep Pediatr.

[35] Murakami H, Tsurusaki Y, Enomoto K (2020). Update of the genotype and phenotype of KMT2D and KDM6A by genetic screening of 100 patients with clinically suspected Kabuki syndrome. Am J Med Genet A.

[36] Froimchuk E, Jang Y, Ge K (2017). Histone H3 lysine 4 methyltransferase KMT2D. Gene.

[37] Butcher DT, Cytrynbaum C, Turinsky AL (2017). CHARGE and Kabuki syndromes: gene-specific DNA methylation signatures identify epigenetic mechanisms linking these clinically overlapping conditions. Am J Hum Genet.

[38] Aref-Eshghi E, Rodenhiser DI, Schenkel LC (2018). Genomic DNA methylation signatures enable concurrent diagnosis and clinical genetic variant classification in neurodevelopmental syndromes. Am J Hum Genet.

[39] Dhar SS, Lee MG (2021). Cancer-epigenetic function of the histone methyltransferase KMT2D and therapeutic opportunities for the treatment of KMT2D-deficient tumors. Oncotarget.

[40] Vogt J, Wagener R, Montesinos-Rongen M (2019). Array-based profiling of the lymphoma cell DNA methylome does not unequivocally distinguish primary lymphomas of the central nervous system from non-CNS diffuse large B-cell lymphomas. Genes Chromosom Cancer.

[41] Wagener R, López C, Kleinheinz K (2018). IG-MYC+ neoplasms with precursor B-cell phenotype are molecularly distinct from Burkitt lymphomas. Blood.

[42] Koelsche C, Schrimpf D, Stichel D (2021). Sarcoma classification by DNA methylation profiling. Nat Commun.

[43] Bens S, Kolarova J, Beygo J (2016). Phenotypic spectrum and extent of DNA methylation defects associated with multilocus imprinting disturbances. Epigenomics.

[44] Aryee MJ, Jaffe AE, Corrada-Bravo H (2014). Minfi: a flexible and comprehensive Bioconductor package for the analysis of Infinium DNA methylation microarrays. Bioinformatics.

[45] Hovestadt V, Zapatka M (2017) Conumee: Enhanced copy-number variation analysis using Illumina DNA methylation arrays. R package version 1.9.0. 10.1093/bioinformatics/btae029 PMC10868300 38244574

[46] Seki M, Nishimura R, Yoshida K (2015). Integrated genetic and epigenetic analysis defines novel molecular subgroups in rhabdomyosarcoma. Nat Commun.

[47] Shern JF, Chen L, Chmielecki J (2014). Comprehensive genomic analysis of rhabdomyosarcoma reveals a landscape of alterations affecting a common genetic axis in fusion-positive and fusion-negative tumors. Cancer Discov.

[48] Kohsaka S, Shukla N, Ameur N (2014). A recurrent neomorphic mutation in MYOD1 defines a clinically aggressive subset of embryonal rhabdomyosarcoma associated with PI3K-AKT pathway mutations. Nat Genet.

[49] Schott DA, Gerver WJM, Stumpel CTRM (2017). Growth hormone therapy in children with Kabuki syndrome: 1-year treatment results. Horm Res Paediatr.

[50] Brioude F, Kalish JM, Mussa A (2018). Expert consensus document: Clinical and molecular diagnosis, screening and management of Beckwith-Wiedemann syndrome: an international consensus statement. Nat Rev Endocrinol.

[51] Robbins KM, Stabley DL, Holbrook J (2016). Paternal uniparental disomy with segmental loss of heterozygosity of chromosome 11 are hallmark characteristics of syndromic and sporadic embryonal rhabdomyosarcoma. Am J Med Genet A.

[52] Kratz CP, Steinemann D, Niemeyer CM (2007). Uniparental disomy at chromosome 11p15.5 followed by HRAS mutations in embryonal rhabdomyosarcoma: lessons from Costello syndrome. Hum Mol Genet.

[53] Walther C, Mayrhofer M, Nilsson J (2016). Genetic heterogeneity in rhabdomyosarcoma revealed by SNP array analysis. Genes Chromosom Cancer.

[54] Ng SB, Bigham AW, Buckingham KJ (2010). Exome sequencing identifies MLL2 mutations as a cause of Kabuki syndrome. Nat Genet.

[55] Lederer D, Grisart B, Digilio MC (2012). Deletion of KDM6A, a histone demethylase interacting with MLL2, in three patients with Kabuki syndrome. Am J Hum Genet.

[56] Miyake N, Mizuno S, Okamoto N (2013). KDM6A point mutations cause Kabuki syndrome. Hum Mutat.

[57] Armstrong L, Abd El Moneim A, Aleck K (2005). Further delineation of Kabuki syndrome in 48 well-defined new individuals. Am J Med Genet A.

[58] Aricò M, Mussolin L, Carraro E (2015). Non-Hodgkin lymphoma in children with an associated inherited condition: a retrospective analysis of the Associazione Italiana Ematologia Oncologia Pediatrica (AIEOP). Pediatr Blood Cancer.

[59] Morice P, Leary A, Creutzberg C (2016). Endometrial cancer. Lancet.

[60] Baranov E, Hornick JL (2020). Soft tissue special issue: fibroblastic and myofibroblastic neoplasms of the head and neck. Head Neck Pathol.

[61] Mentzel T, Pedeutour F (2020) Giant cell fibroblastoma. In: the WHO classification of tumours editorial board (ed) WHO Classification of tumours soft tissue and bone tumours, 5th edn. IARC/Lyon, France, pp 98–99

[62] Bossi D, Cicalese A, Dellino GI (2016). In vivo genetic screens of patient-derived tumors revealed unexpected frailty of the transformed phenotype. Cancer Discov.

[63] Gabriele M, Vitriolo A, Cuvertino S (2021). KMT2D haploinsufficiency in Kabuki syndrome disrupts neuronal function through transcriptional and chromatin rewiring independent of H3K4-monomethylation. bioRxiv.

[64] Maitituoheti M, Keung EZ, Tang M (2020). Enhancer reprogramming confers dependence on glycolysis and IGF signaling in KMT2D mutant melanoma. Cell Rep.

[65] Alam H, Tang M, Maitituoheti M (2020). KMT2D deficiency impairs super-enhancers to confer a glycolytic vulnerability in lung cancer. Cancer Cell.

[66] Lv S, Wen H, Shan X (2019). Loss of KMT2D induces prostate cancer ROS-mediated DNA damage by suppressing the enhancer activity and DNA binding of antioxidant transcription factor FOXO3. Epigenetics.

[67] Skvortsova K, Masle-Farquhar E, Luu P-L (2019). DNA hypermethylation encroachment at CpG Island borders in cancer is predisposed by H3K4 monomethylation patterns. Cancer Cell.

[68] Lv S, Ji L, Chen B (2018). Histone methyltransferase KMT2D sustains prostate carcinogenesis and metastasis via epigenetically activating LIFR and KLF4. Oncogene.

[69] Ford DJ, Dingwall AK (2015). The cancer COMPASS: navigating the functions of MLL complexes in cancer. Cancer Genet.

[70] Rao RC, Dou Y (2015). Hijacked in cancer: the KMT2 (MLL) family of methyltransferases. Nat Rev Cancer.

[71] Bögershausen N, Tsai I-C, Pohl E (2015). RAP1-mediated MEK/ERK pathway defects in Kabuki syndrome. J Clin Invest.

[72] Niemeyer CM (2014). RAS diseases in children. Haematologica.

[73] Smpokou P, Zand DJ, Rosenbaum KN, Summar ML (2015). Malignancy in Noonan syndrome and related disorders. Clin Genet.

[74] Steliarova-Foucher E, Colombet M, Ries LAG (2017). International incidence of childhood cancer, 2001–10: a population-based registry study. Lancet Oncol.

[75] Horibe K, Saito AM, Takimoto T (2013). Incidence and survival rates of hematological malignancies in Japanese children and adolescents (2006–2010): based on registry data from the Japanese Society of Pediatric He lmatology. Int J Hematol.

[76] Kaatsch P (2010). Epidemiology of childhood cancer. Cancer Treat Rev.

[77] Panea RI, Love CL, Shingleton JR (2019). The whole-genome landscape of Burkitt lymphoma subtypes. Blood.

[78] Grande BM, Gerhard DS, Jiang A (2019). Genome-wide discovery of somatic coding and noncoding mutations in pediatric endemic and sporadic Burkitt lymphoma. Blood.

[79] López C, Kleinheinz K, Aukema SM (2019). Genomic and transcriptomic changes complement each other in the pathogenesis of sporadic Burkitt lymphoma. Nat Commun.

[80] Richter J, John K, Staiger AM (2021). Epstein-barr virus status of sporadic Burkitt lymphoma is associated with patient age and mutational features. Br J Haematol.

[81] Thomas N, Dreval K, Gerhard DS (2021). Genetic subgroups inform on pathobiology in adult and pediatric burkitt lymphoma. medRxiv.

[82] Elgaafary S, López C, Nagel I (2021). Molecular characterization of Burkitt lymphoma in the breast or ovary. Leuk Lymphoma.

[83] Pilarowski GO, Cazares T, Zhang L (2020). Abnormal Peyer patch development and B-cell gut homing drive IgA deficiency in Kabuki syndrome. J Allergy Clin Immunol.

[84] Lindsley AW, Saal HM, Burrow TA (2016). Defects of B-cell terminal differentiation in patients with type-1 Kabuki syndrome. J Allergy Clin Immunol.

[85] Lin J-L, Lee W-I, Huang J-L (2015). Immunologic assessment and KMT2D mutation detection in Kabuki syndrome. Clin Genet.

[86] Zhang J, Dominguez-Sola D, Hussein S (2015). Disruption of KMT2D perturbs germinal center B cell development and promotes lymphomagenesis. Nat Med.

[87] Ortega-Molina A, Boss IW, Canela A (2015). The histone lysine methyltransferase KMT2D sustains a gene expression program that represses B cell lymphoma development. Nat Med.

[88] Stagi S, Gulino AV, Lapi E, Rigante D (2016). Epigenetic control of the immune system: a lesson from Kabuki syndrome. Immunol Res.

[89] Lino CNR, Ghosh S (2021). Epstein-barr virus in inborn immunodeficiency—more than infection. Cancers (Basel).

[90] Chmielecki J, Bailey M, He J (2017). Genomic profiling of a large set of diverse pediatric cancers identifies known and novel mutations across tumor spectra. Cancer Res.

[91] Spina V, Bruscaggin A, Cuccaro A (2018). Circulating tumor DNA reveals genetics, clonal evolution, and residual disease in classical Hodgkin lymphoma. Blood.

[92] Reichel J, Chadburn A, Rubinstein PG (2015). Flow sorting and exome sequencing reveal the oncogenome of primary Hodgkin and Reed-Sternberg cells. Blood.

[93] Tiacci E, Ladewig E, Schiavoni G (2018). Pervasive mutations of JAK-STAT pathway genes in classical Hodgkin lymphoma. Blood.

[94] Wienand K, Chapuy B, Stewart C (2019). Genomic analyses of flow-sorted Hodgkin Reed-Sternberg cells reveal complementary mechanisms of immune evasion. Blood Adv.

[95] Desch A-K, Hartung K, Botzen A (2020). Genotyping circulating tumor DNA of pediatric Hodgkin lymphoma. Leukemia.

[96] Haines K, Sarabia SF, Alvarez KR (2019). Characterization of pediatric hepatocellular carcinoma reveals genomic heterogeneity and diverse signaling pathway activation. Pediatr Blood Cancer.

[97] Zhou S-L, Zhou Z-J, Hu Z-Q (2019). Genomic sequencing identifies WNK2 as a driver in hepatocellular carcinoma and a risk factor for early recurrence. J Hepatol.

[98] Cancer Genome Atlas Research Network (2017) Comprehensive and integrative genomic characterization of hepatocellular carcinoma. Cell 169:1327–1341.e23. 10.1016/j.cell.2017.05.046 10.1016/j.cell.2017.05.046 PMC5680778 28622513

[99] Harding JJ, Nandakumar S, Armenia J (2019). Prospective genotyping of hepatocellular carcinoma: clinical implications of next-generation sequencing for matching patients to targeted and immune therapies. Clin Cancer Res.

[100] Zhang J, McCastlain K, Yoshihara H (2016). Deregulation of DUX4 and ERG in acute lymphoblastic leukemia. Nat Genet.

[101] Zaliova M, Stuchly J, Winkowska L (2019). Genomic landscape of pediatric B-other acute lymphoblastic leukemia in a consecutive European cohort. Haematologica.

[102] Waanders E, Gu Z, Dobson SM (2020). Mutational landscape and patterns of clonal evolution in relapsed pediatric acute lymphoblastic leukemia. Blood Cancer Discov.

[103] Li J-F, Dai Y-T, Lilljebjörn H (2018). Transcriptional landscape of B cell precursor acute lymphoblastic leukemia based on an international study of 1,223 cases. Proc Natl Acad Sci USA.

[104] Gröbner SN, Worst BC, Weischenfeldt J (2018). The landscape of genomic alterations across childhood cancers. Nature.

[105] Murphy AJ, Chen X, Pinto EM (2019). Forty-five patient-derived xenografts capture the clinical and biological heterogeneity of Wilms tumor. Nat Commun.

[106] Gadd S, Huff V, Walz AL (2017). A Children’s Oncology Group and TARGET initiative exploring the genetic landscape of Wilms tumor. Nat Genet.

[107] Pugh TJ, Morozova O, Attiyeh EF (2013). The genetic landscape of high-risk neuroblastoma. Nat Genet.

[108] Bellini A, Bessoltane-Bentahar N, Bhalshankar J (2019). Study of chromatin remodeling genes implicates SMARCA4 as a putative player in oncogenesis in neuroblastoma. Int J Cancer.

[109] Sekiguchi M, Seki M, Kawai T (2020). Integrated multiomics analysis of hepatoblastoma unravels its heterogeneity and provides novel druggable targets. NPJ Precis Oncol.

[110] Sumazin P, Chen Y, Treviño LR (2017). Genomic analysis of hepatoblastoma identifies distinct molecular and prognostic subgroups. Hepatology.

[111] Liu J, Gao C, Wang L (2021). Trans-ancestry mutation landscape of hepatoblastoma genomes in children. Front Oncol.

[112] Reddy A, Zhang J, Davis NS (2017). Genetic and functional drivers of diffuse large B cell lymphoma. Cell.

[113] Ramis-Zaldivar JE, Gonzalez-Farré B, Balagué O (2020). Distinct molecular profile of IRF4-rearranged large B-cell lymphoma. Blood.

[114] Pillonel V, Juskevicius D, Ng CKY (2018). High-throughput sequencing of nodal marginal zone lymphomas identifies recurrent BRAF mutations. Leukemia.

[115] Spina V, Khiabanian H, Messina M (2016). The genetics of nodal marginal zone lymphoma. Blood.

[116] Bühler MM, Martin-Subero JI, Pan-Hammarström Q (2021). Towards precision medicine in lymphoid malignancies. J Intern Med.

[117] Ardeshir-Larijani F, Bhateja P, Lipka MB (2018). KMT2D mutation is associated with poor prognosis in non-small-cell lung cancer. Clin Lung Cancer.

[118] Moss TJ, Qi Y, Xi L (2017). Comprehensive genomic characterization of upper tract urothelial carcinoma. Eur Urol.

[119] Sfakianos JP, Cha EK, Iyer G (2015). Genomic characterization of upper tract urothelial carcinoma. Eur Urol.

[120] Audenet F, Isharwal S, Cha EK (2019). Clonal relatedness and mutational differences between upper tract and bladder urothelial carcinoma. Clin Cancer Res.

[121] Dai W, Ko JMY, Choi SSA (2017). Whole-exome sequencing reveals critical genes underlying metastasis in oesophageal squamous cell carcinoma. J Pathol.

[122] Hao J-J, Lin D-C, Dinh HQ (2016). Spatial intratumoral heterogeneity and temporal clonal evolution in esophageal squamous cell carcinoma. Nat Genet.

[123] Northcott PA, Jones DTW, Kool M (2012). Medulloblastomics: the end of the beginning. Nat Rev Cancer.

[124] Northcott PA, Buchhalter I, Morrissy AS (2017). The whole-genome landscape of medulloblastoma subtypes. Nature.

[125] Wong GC-H, Li KK-W, Wang W-W (2020). Clinical and mutational profiles of adult medulloblastoma groups. Acta Neuropathol Commun.

[126] Faundes V, Malone G, Newman WG, Banka S (2019). A comparative analysis of KMT2D missense variants in Kabuki syndrome, cancers and the general population. J Hum Genet.

[127] Cocciadiferro D, Augello B, de Nittis P (2018). Dissecting KMT2D missense mutations in Kabuki syndrome patients. Hum Mol Genet.

[128] Cuvertino S, Hartill V, Colyer A (2020). A restricted spectrum of missense *KMT2D* variants cause a multiple malformations disorder distinct fromKabuki syndrome. Genet Med.

[129] Horak P, Leichsenring J, Goldschmid H (2021). Assigning evidence to actionability: an introduction to variant interpretation in precision cancer medicine. Genes Chromosom Cancer.

[130] Green MR, Kihira S, Liu CL (2015). Mutations in early follicular lymphoma progenitors are associated with suppressed antigen presentation. Proc Natl Acad Sci USA.

[131] Vogelsberg A, Steinhilber J, Mankel B (2020). Genetic evolution of in situ follicular neoplasia to aggressive B-cell lymphoma of germinal center subtype. Haematologica.

[132] Baker SC, Mason AS, Southgate J (2020). Does a novel mutagenic process target KMT2D mutation in the most common first event on the path to bladder cancer?. Eur Urol.

[133] Ma X, Liu Y, Liu Y (2018). Pan-cancer genome and transcriptome analyses of 1,699 paediatric leukaemias and solid tumours. Nature.

[134] Ding X, He M, Chan AWH (2020). Genomic and epigenomic features of primary and recurrent hepatocellular carcinomas. Gastroenterology.

[135] Juhlin CC, Stenman A, Haglund F (2015). Whole-exome sequencing defines the mutational landscape of pheochromocytoma and identifies KMT2D as a recurrently mutated gene. Genes Chromosom Cancer.

[136] Santen GWE, Kriek M, van Attikum H (2012). SWI/SNF complex in disorder: SWItching from malignancies to intellectual disability. Epigenetics.

[137] Holsten T, Bens S, Oyen F (2018). Germline variants in SMARCB1 and other members of the BAF chromatin-remodeling complex across human disease entities: a meta-analysis. Eur J Hum Genet.

[138] Shern JF, Yohe ME, Khan J (2015). Pediatric rhabdomyosarcoma. Crit Rev Oncog.

[139] Brodeur GM, Nichols KE, Plon SE (2017). Pediatric cancer predisposition and surveillance: an overview, and a tribute to Alfred G. Knudson Jr. Clin Cancer Res.

[140] Saade-Lemus S, Degnan AJ, Acord MR (2019). Whole-body magnetic resonance imaging of pediatric cancer predisposition syndromes: special considerations, challenges and perspective. Pediatr Radiol.

[141] Greer M-LC (2018). Imaging of cancer predisposition syndromes. Pediatr Radiol.

[142] Paramathas S, Guha T, Pugh TJ (2020). Considerations for the use of circulating tumor DNA sequencing as a screening tool in cancer predisposition syndromes. Pediatr Blood Cancer.

[143] Hettmer S, Archer NM, Somers GR (2014). Anaplastic rhabdomyosarcoma in TP53 germline mutation carriers. Cancer.

[144] Shenoy A, Alvarez E, Chi Y-Y (2020). The prognostic significance of anaplasia in childhood rhabdomyosarcoma: a report from the Children’s Oncology Group. Eur J Cancer.

[145] Sciot R, Gerosa C, Fanni D, Sciot R, Gerosa C, Faa G (2020). Skeletal muscle tumors BT. Adipocytic, vascular and skeletal muscle tumors: a practical diagnostic approach.

