# Supplementary material for: Molecular characterization of an embryonal rhabdomyosarcoma occurring in a patient with Kabuki syndrome: report and literature review in the light of tumor predisposition syndromes
Source: Fam Cancer. 2022 Jul 19;22(1):103–18. doi: 10.1007/s10689-022-00306-z (PMC9829644; doi:10.1007/s10689-022-00306-z)
Supplement: Supplementary file 1 — Supplementary file1 (PDF 502 kb) [file 10689_2022_306_MOESM1_ESM.pdf]

Supporting Data to

Molecular Characterization of an Embryonal Rhabdomyosarcoma Occurring in a Patient with Kabuki Syndrome.

Report and Literature Review in the Light of Tumor Predisposition Syndromes

Sietse M. Aukema[1,*], Selina Glaser[2,*], Mari F. C. M. van den Hout[3],

Sonja Dahlum[2], Marinus J. Blok[1], Morten Hillmer[2], Julia Kolarova[2],

Raf Sciot[4], Dina A. Schott[1,5], Reiner Siebert[2], Constance T. R. M. Stumpel[1,6]

*Sietse M. Aukema and Selina Glaser are joined first authors.

.

Supporting data

Supporting methods

Literature review The PubMed (ncbi.nlm.nih.gov/) based search included search terms "Kabuki and cancer", "Kabuki and malignancy", "Kabuki and tumor" as well as "*KMT2D* and cancer", "*KMT2D* and malignancy" and "*KMT2D* and tumor". In addition, the references in (case) reports of patients with Kabuki syndrome and a concomitant malignancy were screened for relevant publications. Also articles including the Supporting Data reporting on large series of patients with Kabuki syndrome were explored for patients with a malignancy. In the case no *KMT2D* or *KDM6A* variant was reported, the clinical features (photographs, clinical characteristics) were assessed for substantiation of the diagnosis of Kabuki syndrome by two investigators (S.M.A. and C.T.R.M.S.). With the Kabuki syndrome international consensus diagnostic criteria as guideline [1], the clinical diagnosis in those cases was scored as following: "(clinical features compatible with) Kabuki syndrome", "clinical features not compatible with Kabuki syndrome" or "insufficient information for assessment of the diagnosis". Only high-grade or intermediate-grade malignancies were included. E.g., patients with Kabuki syndrome and Becker naevus [2], pilomatrixoma [3, 4] or non-monoclonal lymphoproliferation [5] were excluded. In addition one recently published patient with Kabuki syndrome and multiple endocrine neoplasia type 2 A (MEN2A) caused by a pathogenic *RET* mutation who developed a pheochromocytoma was not included in the analysis (as if the pheochromocytoma was benign or malignant was not reported and most MEN2 related pheochromocytomas have a low risk for malignancy [6, 7]) [8]. Two patients with pheochromocytoma [9] and one with severe aplastic anaemia [10], who had constitutional missense *KMT2D* variants were not included as these patients either lacked any Kabuki-syndrome related features [9] or lacked a (clear) Kabuki phenotype (dysmorphism and intellectual disability were absent) [10]. Also patients with rare, non-truncating *KMT2D* germline variants and B-cell neoplasms were excluded [11]. Along the same lines, (pediatric) patients with Hodgkin lymphoma [12] and neuroblastoma [13] with

missense germline *KMT2D* variants but without any mentioning of Kabuki syndrome were not included. Subsequently we tried – based on the data presented in the individual manuscripts - to evaluate if the clinicopathologic and molecular features were typical for that malignancy type in the general population.

DNA-methylation : Analysis of the genome-wide methylation pattern was performed as previously described [14, 15]. In brief, ≈ 500 ng of genomic DNA isolated from fresh frozen tissue was subjected to bisulfite conversion using the EZ DNA Methylation kit (ZymoResearch, Irvine, CA, USA) according to manufacturers' instructions. Subsequently, the bisulfit converted DNA was hybridized on Infinium® MethylationEPIC (EPIC) BeadChip (Illumina Inc., San Diego USA) according to manufacturers' protocol. Raw methylation data was processed and normalised using the GenomeStudio software (v2011.1; methylation module 1.9.0; Illumina) applying the default settings. For data normalisation, the intrinsic controls present on the array were used. Subsequently, rs loci, loci on gonosomes and loci with a detection p value > 0.01 were excluded from further analysis. For molecular classification the "DKFZ sarcoma classifier" (version v12.2) available on www.molecularneuropathology.org/msp/ was applied [16]. DNA methylation data of five controls (blood), hybridized on the Human Methylation 450k BeadChip (450k array) and processed as already described in Bens et al., were used for comparison [17]. DNA methylation values corresponding to the imprinted regions H19 / imprinting center 1 (IC1) / differentially methylated region 1 (DMR1) and KCNQ1/KCNQ1OT1 imprinting center 2 (IC2) / differentially methylated region 2 (DMR2) were extracted from the 850k (n= 82 CpGs) and 450k (n=75 CpGs) data and visually compared. For copy number variant (CNV) analysis of the methylation data the R-package minfi (version 1.40.0) was used for normalisation and subsequently analysed with the package conumee (version 1.24.0)[18, 19].

Exome-sequencing (ES) : ES was performed on ≈ 50 ng of genomic DNA isolated from fresh frozen tissue using the NextSeq High Output Kit v2.5 (300 cycles; Illumina: Nextera™ Exome Kit) and sequenced on a NextSeq platform. Data was analysed with Varvis-Software Version 1.18.4 (Limbus Medical Technologies GmbH, Rostock). Genome build GRCh37/hg19 was

used. Exome data were filtered for a virtual panel of cancer predisposition genes with a total of 107 genes analyzed (according to the TruSightCancer Panel Illumina, San Diego, Ca., USA; 95 genes, Supporting Table S1). In addition *KMT2D*, *KDM6A* as well as 10 genes recurrently mutated in embryonal and/or fusion negative rhabdomyosarcoma were analysed [20–22].

Supporting references Table 1 and Supporting Table S4

The Supporting references for the common/general (clinical)presentation for the reported tumor entities include: embryonal rhabdomyosarcoma [23, 24], synovial sarcoma [25], low-grade fibromyxoid sarcoma [26, 27], giant cell fibroblastoma [28, 29], desmoid fibromatosis [29–32], Burkitt lymphoma [33, 34], Hodgkin lymphoma [35], pre-B-ALL [36], Wilms tumor / nephroblastoma [37, 38], neuroblastoma [37–39], hepatoblastoma [37, 38, 40], ependymoma [41], hepatocellular adenoma [42, 43] hepatocellular carcinoma [43, 44] and endometrial cancer [45].

Legends to Supporting Figures

Supporting Figure S1

Heatmap of the Beckwith-Wiedemann imprinted locus on chromosome 11p15 showing methylation of the rhabdomyosarcoma of the patient (orange) compared to the 5 controls (blood) (grey). A DNA methylation value (Beta-value) of 0.5 was set as normal, a value of 0.0 indicates hypo- and 1.0 hypermethylation.

Supporting Figure S2

Screenshot from Integrative Genomics Viewer (IGV) of Sanger sequence of the position of the germline variant c.2558_2559delCT in Exon 10 in tumor material. Upper panel, reverse sequence and lower panel, forward sequence. Many of the nucleotide peaks are overlapping but in case two non-overlapping peaks are present at the same nucleotide position almost exclusively the wild-type sequence (depicted on top in green) has higher peaks compared to the mutated sequence which is suggestive of gain of the wild-type and not the mutated allele in the trisomy 12 (figure 1E).

Supporting Figure S1

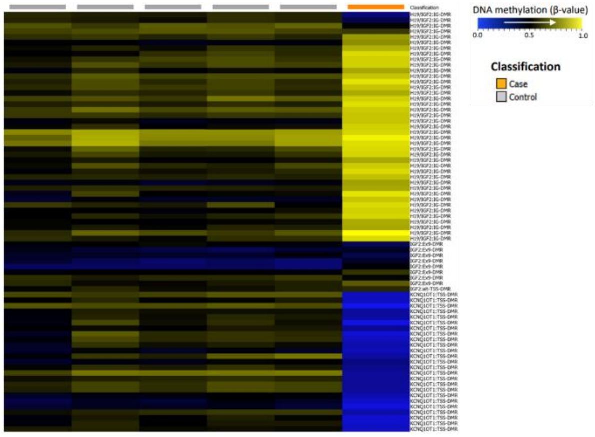

Supporting Figure S2

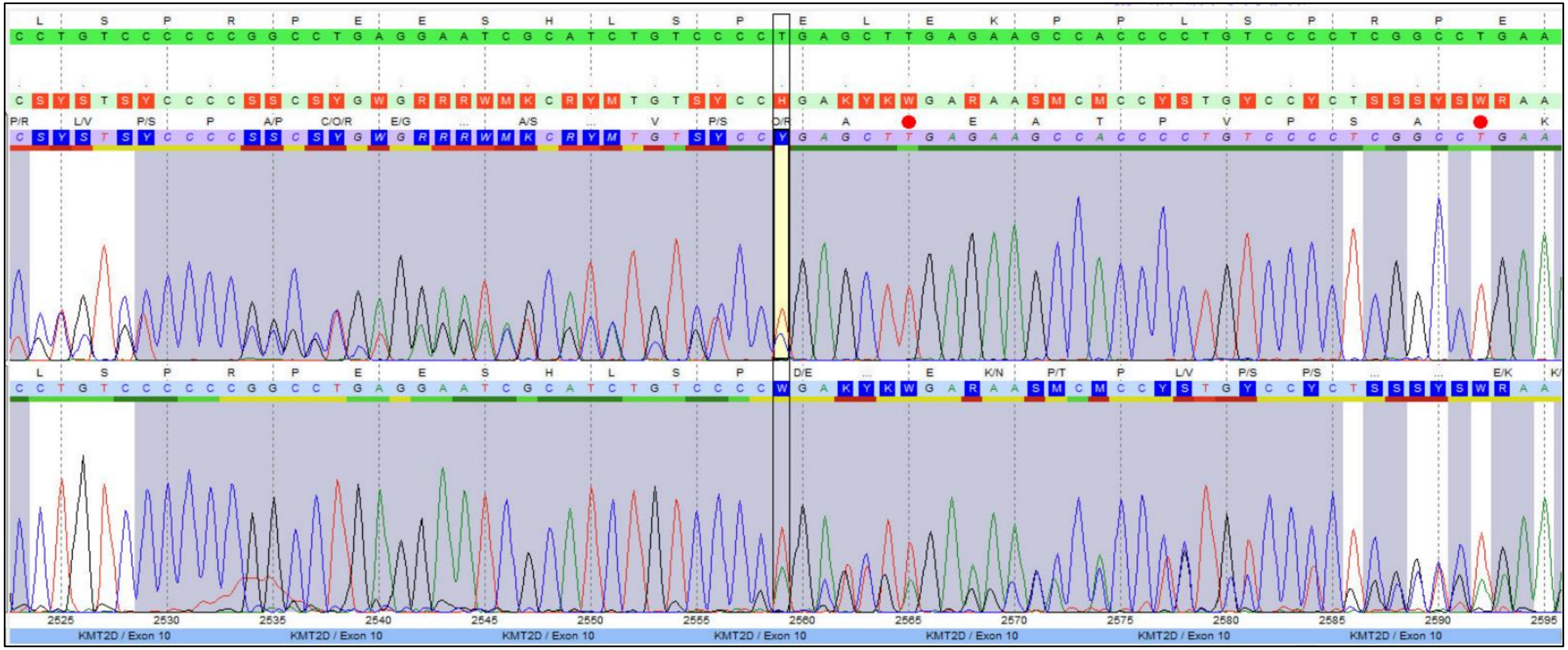

Supporting Table S1

Gene list of the virtual panel including all 107 analysed tumor predisposition, Kabuki syndrome and sarcoma related genes.

AIP	*CDH1*	*ERCC3*	*FANCL*	*KRAS*	*PALB2*	*RECQL4*	*TMEM127*
ALK	*CDK4*	*ERCC4*	*FANCM*	*MAX*	*PHOX2B*	*RET*	*TP53*
APC	*CDKN1C*	*ERCC5*	*FBXW7*	*MEN1*	*PIK3CA*	*RHBDF2*	*TSC1*
ARID1A	*CDKN2A*	*EXT1*	*FGFR4*	*MET*	*PMS1*	*RUNX1*	*TSC2*
ATM	*CEBPA*	*EXT2*	*FH*	*MLH1*	*PMS2*	*SBDS*	*VHL*
BAP1	*CEP57*	*EZH2*	*FLCN*	*MSH2*	*PPM1D*	*SDHAF2*	*WRN*
BCOR	*CHEK2*	*FANCA*	*GAB1*	*MSH6*	*PRF1*	*SDHB*	*WT1*
BLM	*CYLD*	*FANCB*	*GATA2*	*MUTYH*	*PRKAR1A*	*SDHC*	*XPA*
BMPR1A	*DDB2*	*FANCC*	*GPC3*	*MYOD1*	*PTCH1*	*SDHD*	*XPC*
BRCA1	*DICER1*	*FANCD2*	*HNF1A*	*NBN*	*PTEN*	*SLX4*	
BRCA2	*DIS3L2*	*FANCE*	*HRAS*	*NF1*	*PTPN11*	*SMAD4*	
BRIP1	*EGFR*	*FANCF*	*KDM6A*	*NF2*	*RAD51C*	*SMARCB1*	
BUB1B	*EPCAM*	*FANCG*	*KIT*	*NRAS*	*RAD51D*	*STK11*	
CDC73	*ERCC2*	*FANCI*	*KMT2D*	*NSD1*	*RB1*	*SUFU*	

**Supporting Table S2: primer sequences used for Sanger sequencing of the germline variant in tumor DNA.**	
KMT2D_Ex_10_P1_F	**GTAGCGCGACGGCCAGTATTGAGGAATTGCACCTGTCC**
KMT2D_Ex_10_P1_R	**CAGGGCGCAGCGATGACGCAAAAGAAGGGCTCTTAGATTAGATG**

Supporting Table S3: Identified variants in the ERMS-tumor tissue by exome sequencing[a]											
								Variant predictions[b]		
**Variant position and description**	**Alt Func**	**Cov / VAF**	**Gene**	**Transcript**	**c.DNA**	**AA Change**	**COSMIC/ SOM**	**SIFT**	**PP**	**CADD**
Chr13(GRCh37):g.103510688C>G	MV	25/76	*ERCC5*	NM_000123.4	c.592C>G	p.(Pro198Ala)	No	T	PD	20.8
Chr17(GRCh37):g.7579472G>C	MV	50[d]	*TP53*	NM_000546.5	c.215C>G[d]	p.(Pro72Arg)	YES/YES	T	B	9.2
**Variants in *KMT2D***										
Chr12(GRCh37):g.49444907_49444908del[c]	Fs	NA	*KMT2D*	ENST00000301067.7	c.2558_2559del	p.Pro853Argfs*3	NO	NA	NA	23.3
Chr12(GRCh37):g.49439659C>T	Intron	88[d]	*KMT2D*	ENST00000301067.7	c.4741+44G>A[d]	p.?	NO	NA	NA	NA
Chr12(GRCh37):g.49424534G>A	SYN	35/49	*KMT2D*	ENST00000301067.7	c.13689C>T	p.Pro4563(=)	YES/YES	NA	NA	NA
**Variants in *KDM6A***										
ChrX(GRCh37):g.44938563G>A	SYN	56[d]	*KDM6A*	ENST00000377967.4	c.3111G>A[d]	p.Gln1037(=)	YES/YES	NA	NA	NA

AA, amino acid ; Alt, alteration/altered ; CADD, Combined Annotation Dependent Depletion score ; Chr, chromosome ; COSMIC, Catalogue Of Somatic Mutations In Cancer ; Cov, coverage at nucleotide position ; ERMS, embryonal rhabdomyosarcoma ; Fs, frameshift ; Func, function ; MV, missense variant ; NA, not applicable ; PP, Polymorphism Phenotyping ; SIFT, Sorting Intolerant from Tolerant ; SOM, somatic origin of the variant (once) confirmed in COSMIC-database ; SYN, synonymous variant ; VAF, variant allele frequency (%).
[a]missense variants are predicted to have moderate impact, truncating variants high-impact. Low-impact variants are not included. The variant in *ERCC5* is a class 3 variant, the truncating *KMT2D* variant class 5. All other variants probably benign/likely benign.
[b]For synonymous variants no variant predictions performed. For splice variants SIFT and PolyPhen predictions are not applicable.
[c]nucleotide position of germline variant insufficiently covered with exome sequencing, determined by Sanger-sequencing.
[d]Homozygous.

Supporting Table S4: Summary of literature review of patients with Kabuki syndrome and a malignancy with insufficient information									
**Pt**	**Sex, age**	***KMT2D*/*KDM6A* variant**	**Clinical diagnosis (variant neg/ NA)[a]**	**Malignancy[b]**	**Histopathological features**	**Molecular tumor features**	**Typical (clinical) presentation (refs)?[c]**	**Predisposing factors?[s]**	**Reference case**
**Hematologic malignancies (n=2)**									
S1	M (5)	NR	Insufficient information	Burkitt lymphoma	NR	NR	Yes. Typical age and gender.	No (potential) predisposing factors reported other than Kabuki syndrome.	[46]
S2[e]	NR, childhood	NR	Insufficient information	NHL	NR	NR	NA	NA	[47]
**Embryonal tumours (n=1)**									
S3[e]	NR	NR	(compatible with) Kabuki syndrome[f]	Neuroblastoma	NR	NR	NA	NA	[48]

M, male ; NA, not available ; NHL, non-Hodgkin lymphoma ; NR, not reported.
[a]Clinical diagnosis based on assessment of clinical features presented in the individual manuscripts by the author's of the present study, see the "supplementary materials and method's" section.
[b]Diagnosis, clinico- and histopathologic features as provided in the original manuscript. These may not fulfil the present World Health Organisation (WHO)-criteria for the respective tumors.
[c]References for common/general (clinical) presentation of individual tumor entities in Supporting References.
[d]Includes (other)potential predisposing (genetic) factors for the reported malignancy
[e]Insufficient information to include in literature review and/or discussion. Overlap with other published cases can not be excluded.
[f]Patients in the included manuscript have a clinical diagnosis (compatible with) Kabuki syndrome but for the individual patients, including the patient with neuroblastoma, no individual patient characteristics are provided. Due to the limited information provided for this patient this patient was excluded from the literature review.

Supporting Table S5: Summary of oncological characteristics, therapy and outcome of patients with Kabuki syndrome and a malignancy[a]							
**Pt**	**Sex, age**	**Malignancy[c]**	**Relapse, SMN, bilateral-/multifocal or meta-synchronous cancers?**	**Therapy**	**Excessive chemotherapy toxicity?**	**Outcome**	**Ref. case**
**Bone- and soft-tissue tumors (n=5)**							
1	F (10)	Embryonal rhabdomyosarcoma	No	Chemotherapy, surgery	Neutropenic sepsis[b]	Deceased after sepsis post-chemotherapy	*this study*
2	F (16)	Synovial sarcoma	Local relapse +4 Mo	Chemotherapy, surgery, radiotherapy	Not reported but no specific information regarding presence/absence of toxicity to chemotherapy.	Deceased within weeks after relapse due to progression	[49]
3	F (10)	Low-grade fibromyxoid sarcoma	No	Surgery	Not applicable (surgery)	No recurrence, +16 Mo after resection	[50]
4	F (10 & 12)	Giant cell fibroblastoma[d]	Recurrence +19 Mo after surgical resection	10 yrs: surgery<br>12 yrs: surgery	Not applicable (surgery)	No relapse at +6 Mo after resection of second tumor	[51]
5	F (10)	Aggressive desmoid fibromatosis	No	Surgery	Not applicable (surgery)	No (long term) follow-up reported	[52]
**Hematologic malignancies (n=5)**							
6	M (5)	Burkitt lymphoma	No	Surgery, chemotherapy	Not reported but no specific information regarding presence/absence of toxicity to chemotherapy	Complete remission, no evidence of disease +11.5 Yr	[53]
7	M (3)	Burkitt lymphoma	No	Chemotherapy	Not reported but no specific information regarding presence/absence of toxicity to chemotherapy	Complete remission followed by maintenance therapy, no relapse +3 Yr	[54]
8	M (3)	Burkitt lymphoma	NA	NA	NA	NA	[55]
9	M (≥32)	Hodgkin lymphoma	No[e]	Chemotherapy	Not reported but no specific information regarding toxicity response to chemotherapy	Complete remission	[56]
10	F (2)	Pre-B-ALL	No	Chemotherapy	Repetitive seizures[f]	Complete remission, +14 Mo after diagnosis (under maintenance therapy)	[57]

Pt	Sex, age	Malignancy[c]	Relapse, SMN, bilateral-/multifocal or meta-synchronous cancers?	Therapy	Excessive chemotherapy toxicity?	Outcome	Ref. case
**Embryonal tumors (n=4)**							
11	F (3)	Wilms tumor / nephroblastoma	No	Surgery, chemotherapy	Liver dysfunction (grade 3) and seizures (grade 2) requiring dose reductions and interval adjustments. No late effects after chemotherapy	Disease-free +18 Mo after diagnosis	[58]
12	F (0)	Neuroblastoma	No	Surgery	Not applicable (surgery)	Complete remission, +18 Mo after diagnosis	[59]
13	F (NR)	Neuroblastoma	NA	NA	NA	NA	[60]
14	M (6)	Fetal-type hepatoblastoma	No	Chemotherapy and surgery	No	Complete remission, +2 Yr after end of treatment	[59]
**Other (n=4)**							
15	F (23)	Ependymoma	No	Surgery	Not applicable (surgery)	Disease free, +14 Mo after diagnosis	[61]
16	F (15)	Hepatocellular carcinoma	No[g]	Surgery (liver transplantation)	Not applicable (surgery)	NA	[62]
17	F (16)	Carcinoma, unknown primary origin	No	Chemotherapy	Sepsis following febrile neutropenia[b] after chemotherapy	Progression. Deceased after sepsis, respiratory failure and cancerous lymphangiosis	[63]
18	F (≤31)[h]	Endometrial cancer	NA	NA	NA	NA	[64]

B-ALL, B-cell acute lymphoblastic leukemia ; F, female ; M, male ; Mo, months ; NA, not available ; NR, not reported ; Pt, patient ; SMN, second malignant neoplasms ; Yr, year.
[a]Patient S1 with Burkitt lymphoma with insufficient clinical information regarding Kabuki syndrome is not present in this table but is included in Supporting Table S4.
[b]Absolute neutrophil count not provided. [c]Diagnosis as provided in the original manuscript. This may not fulfil the present World Health Organisation (WHO) criteria for the respective tumor. [d]Initial tumor diagnosed as spindle cell hemangioma, 2nd tumor at same site as giant cell fibroblastoma. [e]Extensive lipomatosis, benign tumor. [f]State of consciousness during seizures and/or if medication was given for seizure control not provided. [g]Multifocal hepatic adenomas, single high-grade neoplasm (hepatocellular carcinoma). [h]Age at last examination.

Supporting References

1. Adam MP, Banka S, Bjornsson HT, et al (2019) Kabuki syndrome: international consensus diagnostic criteria. J Med Genet 56:89–95. https://doi.org/10.1136/jmedgenet-2018-105625
2. Cuesta L, Betlloch I, Toledo F, et al (2011) Kabuki syndrome: a new case associated with Becker nevus. Dermatol Online J 17:1
3. Hamahata A, Kamei W, Ishikawa M, et al (2013) Multiple pilomatricomas in Kabuki syndrome. Pediatr Dermatol 30:253–255. https://doi.org/10.1111/j.1525-1470.2011.01718.x
4. Bernier F-E, Schreiber A, Coulombe J, et al (2017) Pilomatricoma Associated with Kabuki Syndrome. Pediatr Dermatol 34:e26–e27. https://doi.org/10.1111/pde.13014
5. Marzollo A, Colavito D, Sartori S, et al (2018) Cerebral Lymphoproliferation in a Patient with Kabuki Syndrome. J Clin Immunol 38:475–477
6. Guo Z, Lloyd R v (2015) Pheochromocytomas and Paragangliomas: An Update on Recent Molecular Genetic Advances and Criteria for Malignancy. Advances in Anatomic Pathology 22:
7. Amodru V, Taieb D, Guerin C, et al (2020) MEN2-related pheochromocytoma: current state of knowledge, specific characteristics in MEN2B, and perspectives. Endocrine 69:496–503. https://doi.org/10.1007/s12020-020-02332-2
8. Park E, Kim M-S, Noh ES, et al (2022) Multiple endocrine neoplasia type 2 and autoimmune polyendocrine syndromes (type 1 diabetes mellitus and Graves' disease) in a 16-year-old male with Kabuki syndrome. Endocrine Journal advpub: https://doi.org/10.1507/endocrj.EJ22-0084
9. Juhlin CC, Stenman A, Haglund F, et al (2015) Whole-exome sequencing defines the mutational landscape of pheochromocytoma and identifies KMT2D as a recurrently mutated gene. Genes Chromosomes Cancer 54:542–554. https://doi.org/10.1002/gcc.22267
10. Tamura S, Kosako H, Furuya Y, et al (2021) A Patient with Kabuki Syndrome Mutation Presenting with Very Severe Aplastic Anemia. Acta Haematol 1–8
11. Mosquera Orgueira A, Cid López M, Peleteiro Raíndo A, et al (2021) Detection of Rare Germline Variants in the Genomes of Patients with B-Cell Neoplasms. Cancers (Basel) 13:. https://doi.org/10.3390/cancers13061340
12. Desch A-K, Hartung K, Botzen A, et al (2020) Genotyping circulating tumor DNA of pediatric Hodgkin lymphoma. Leukemia 34:151–166. https://doi.org/10.1038/s41375-019-0541-6
13. Bellini A, Bessoltane-Bentahar N, Bhalshankar J, et al (2019) Study of chromatin remodeling genes implicates SMARCA4 as a putative player in oncogenesis in neuroblastoma. Int J Cancer 145:2781–2791. https://doi.org/10.1002/ijc.32361
14. Wagener R, López C, Kleinheinz K, et al (2018) IG-MYC+ neoplasms with precursor B-cell phenotype are molecularly distinct from Burkitt lymphomas. Blood 132:2280. https://doi.org/10.1182/blood-2018-03-842088
15. Vogt J, Wagener R, Montesinos-Rongen M, et al (2019) Array-based profiling of the lymphoma cell DNA methylome does not unequivocally distinguish primary lymphomas of the central

nervous system from non-CNS diffuse large B-cell lymphomas. Genes Chromosomes Cancer 58:66–69. https://doi.org/10.1002/gcc.22687

16. Koelsche C, Schrimpf D, Stichel D, et al (2021) Sarcoma classification by DNA methylation profiling. Nat Commun 12:498. https://doi.org/10.1038/s41467-020-20603-4

17. Bens S, Kolarova J, Beygo J, et al (2016) Phenotypic spectrum and extent of DNA methylation defects associated with multilocus imprinting disturbances. Epigenomics 8:801–816. https://doi.org/10.2217/epi-2016-0007

18. Hovestadt V, Zapatka M (2017) Conumee: Enhanced copy-number variation analysis using Illumina DNA methylation arrays. R package version 1.9.0.

19. Aryee MJ, Jaffe AE, Corrada-Bravo H, et al (2014) Minfi: a flexible and comprehensive Bioconductor package for the analysis of Infinium DNA methylation microarrays. Bioinformatics 30:1363–1369. https://doi.org/10.1093/bioinformatics/btu049

20. Kohsaka S, Shukla N, Ameur N, et al (2014) A recurrent neomorphic mutation in MYOD1 defines a clinically aggressive subset of embryonal rhabdomyosarcoma associated with PI3K-AKT pathway mutations. Nat Genet 46:595–600. https://doi.org/10.1038/ng.2969

21. Seki M, Nishimura R, Yoshida K, et al (2015) Integrated genetic and epigenetic analysis defines novel molecular subgroups in rhabdomyosarcoma. Nat Commun 6:7557. https://doi.org/10.1038/ncomms8557

22. Shern JF, Chen L, Chmielecki J, et al (2014) Comprehensive genomic analysis of rhabdomyosarcoma reveals a landscape of alterations affecting a common genetic axis in fusion-positive and fusion-negative tumors. Cancer Discov 4:216–231. https://doi.org/10.1158/2159-8290.CD-13-0639

23. Rudzinski ER (2020) Embryonal rhabdomyosarcoma. In: WHO Classification of Tumours Soft Tissue and Bone Tumours, 5th Edition. IARC/Lyon, France, pp 201–204

24. Skapek SX, Ferrari A, Gupta AA, et al (2019) Rhabdomyosarcoma. Nat Rev Dis Primers 5:1. https://doi.org/10.1038/s41572-018-0051-2

25. Suurmeijer A, Ladanyi M, Nielsen T (2020) Synovial sarcoma. In: WHO Classification of Tumours Soft Tissue and Bone Tumours, 5th edition. IARC, Lyon, France, pp 290–293

26. Doyle L, Mertens F (2020) Low-grade fibromyxoid sarcoma. In: WHO Classification of Tumours Soft Tissue and Bone Tumours, 5th Edition. IARC/Lyon, France, pp 127–129

27. Scheer M, Vokuhl C, Veit-Friedrich I, et al (2020) Low-grade fibromyxoid sarcoma: A report of the Cooperative Weichteilsarkom Studiengruppe (CWS). Pediatr Blood Cancer 67:e28009. https://doi.org/10.1002/pbc.28009

28. Mentzel T, Pedeutour F (2020) Giant cell fibroblastoma. In: WHO Classification of Tumours Soft Tissue and Bone Tumours, 5th Edition. IARC/Lyon, France, pp 98–99

29. Baranov E, Hornick JL (2020) Soft Tissue Special Issue: Fibroblastic and Myofibroblastic Neoplasms of the Head and Neck. Head Neck Pathol 14:43–58. https://doi.org/10.1007/s12105-019-01104-3

30. Fritchie K, Crago A, van de Rijn M (2020) Desmoid fibromatosis. In: WHO Classification of Tumours Soft Tissue and Bone Tumours, 5th Edition. IARC/Lyon, France, pp 93–95

31. Peña S, Brickman T, StHilaire H, Jeyakumar A (2014) Aggressive fibromatosis of the head and neck in the pediatric population. Int J Pediatr Otorhinolaryngol 78:1–4. https://doi.org/10.1016/j.ijporl.2013.10.058

32. Coffin CM, Alaggio R (2012) Fibroblastic and myofibroblastic tumors in children and adolescents. Pediatric and developmental pathology : the official journal of the Society for Pediatric Pathology and the Paediatric Pathology Society 15:127–180. https://doi.org/10.2350/10-12-0944-PB.1

33. Leoncini L, Campo E, Stein H, et al (2017) Burkitt lymphoma. In: WHO Classification of Tumours of Hematopoietic and Lymphoid Tissues, 4th revised edition. IARC/Lyon, France, pp 330–334

34. López C, Kleinheinz K, Aukema SM, et al (2019) Genomic and transcriptomic changes complement each other in the pathogenesis of sporadic Burkitt lymphoma. Nature Communications 10:1459. https://doi.org/10.1038/s41467-019-08578-3

35. Stein H, Pileri S, Weiss L, et al (2017) Hodgkin lymphomas: Introduction. In: WHO Classification of Tumours of Hematopoietic and Lymphoid Tissues, 4th Ed. International Agency for Research on Cancer (IARC), Lyon, pp 424–430

36. Borowitz MJ, Chan JKC, Downing JR, et al (2017) B-lymphoblastic leukaemia/lymphoma, not otherwise specified (NOS). In: WHO Classification of Tumours of Hematopoietic and Lymphoid Tissues, 4th Ed. International Agency for Research on Cancer (IARC), Lyon, pp 200–202

37. Allen-Rhoades W, Whittle SB, Rainusso N (2018) Pediatric Solid Tumors of Infancy: An Overview. Pediatr Rev 39:57–67. https://doi.org/10.1542/pir.2017-0057

38. Davenport KP, Blanco FC, Sandler AD (2012) Pediatric malignancies: neuroblastoma, Wilm's tumor, hepatoblastoma, rhabdomyosarcoma, and sacroccygeal teratoma. Surg Clin North Am 92:745–67, x. https://doi.org/10.1016/j.suc.2012.03.004

39. Maris JM (2010) Recent advances in neuroblastoma. N Engl J Med 362:2202–2211. https://doi.org/10.1056/NEJMra0804577

40. Paradis V, Saxena R, Quaglia A Hepatoblastoma. In: The WHO Classification of Tumours Editorial Board (ed) WHO Classification of Tumours Online, Digestive system tumours, 5th Ed. International Agency for Research on Cancer (IARC), Lyon

41. Gerstner ER, Pajtler KW (2018) Ependymoma. Semin Neurol 38:104–111. https://doi.org/10.1055/s-0038-1636503

42. Paradis V, Bioulac-Sage P, Kakar S, Nault J Hepatocellular adenoma. In: The WHO Classification of Tumours Editorial Board (ed) WHO Classification of Tumours Online, Digestive system tumours, 5th Ed. International Agency for Research on Cancer (IARC), Lyon

43. Ng K, Mogul DB (2018) Pediatric Liver Tumors. Clin Liver Dis 22:753–772. https://doi.org/10.1016/j.cld.2018.06.008

44. Schirmacher P, Torbenson M, Park Y, et al Hepatocellular carcinoma. In: The WHO Classification of Tumours Editorial Board (ed) WHO Classification of Tumours Online, Digestive system tumours, 5th Ed. International Agency for Research on Cancer (IARC), Lyon

45. Morice P, Leary A, Creutzberg C, et al (2016) Endometrial cancer. Lancet 387:1094–1108. https://doi.org/10.1016/S0140-6736(15)00130-0

46. Attarbaschi A, Carraro E, Abla O, et al (2016) Non-Hodgkin lymphoma and pre-existing conditions: spectrum, clinical characteristics and outcome in 213 children and adolescents. Haematologica 101:1581–1591. https://doi.org/10.3324/haematol.2016.147116

47. Aricò M, Mussolin L, Carraro E, et al (2015) Non-Hodgkin lymphoma in children with an associated inherited condition: A retrospective analysis of the Associazione Italiana Ematologia Oncologia Pediatrica (AIEOP). Pediatric Blood & Cancer 62:1782–1789. https://doi.org/https://doi.org/10.1002/pbc.25565

48. Armstrong L, Abd El Moneim A, Aleck K, et al (2005) Further delineation of Kabuki syndrome in 48 well-defined new individuals. Am J Med Genet A 132A:265–272. https://doi.org/10.1002/ajmg.a.30340

49. Casanova M, Selicorni A, Ferrari A (2011) Cancer predisposition in children with Kabuki syndrome. Am J Med Genet A 155A:1504

50. Shahdadpuri R, O'Meara A, O'Sullivan M, Reardon W (2008) Low-grade fibromyxoid sarcoma: yet another malignancy associated with Kabuki syndrome. Clin Dysmorphol 17:199–202. https://doi.org/10.1097/MCD.0b013e3282f5f4e3

51. Karagianni P, Lambropoulos V, Stergidou D, et al (2016) Recurrent giant cell fibroblastoma: Malignancy predisposition in Kabuki syndrome revisited. Am J Med Genet A 170A:1333–1338. https://doi.org/10.1002/ajmg.a.37584

52. Scala M, Morana G, Sementa AR, et al (2019) Aggressive desmoid fibromatosis in Kabuki syndrome: Expanding the tumor spectrum. Pediatr Blood Cancer 66:e27831

53. de Billy E, Strocchio L, Cacchione A, et al (2019) Burkitt lymphoma in a patient with Kabuki syndrome carrying a novel KMT2D mutation. Am J Med Genet A 179:113–117. https://doi.org/10.1002/ajmg.a.60674

54. Ijichi O, Kawakami K, Matsuda Y, et al (1996) A case of Kabuki make-up syndrome with EBV+Burkitt's lymphoma. Acta Paediatr Jpn 38:66–68. https://doi.org/10.1111/j.1442-200x.1996.tb03439.x

55. Cheon CK, Sohn YB, Ko JM, et al (2014) Identification of KMT2D and KDM6A mutations by exome sequencing in Korean patients with Kabuki syndrome. J Hum Genet 59:321–325. https://doi.org/10.1038/jhg.2014.25

56. Kaiwar C, Kruisselbrink TM, Kudva YC, et al (2019) Exome sequencing confirms diagnosis of kabuki syndrome in an-adult with hodgkin lymphoma and unusually severe multisystem phenotype. Clin Immunol 207:55–57. https://doi.org/10.1016/j.clim.2018.09.013

57. Scherer S, Theile U, Beyer V, et al (2003) Patient with Kabuki syndrome and acute leukemia. Am J Med Genet A 122A:76–79. https://doi.org/10.1002/ajmg.a.20261

58. Teranishi H, Koga Y, Nakashima K, et al (2018) Cancer Management in Kabuki Syndrome: The First Case of Wilms Tumor and a Literature Review. J Pediatr Hematol Oncol 40:391–394. https://doi.org/10.1097/MPH.0000000000001111

59. Tumino M, Licciardello M, Sorge G, et al (2010) Kabuki syndrome and cancer in two patients. Am J Med Genet A 152A:1536–1539. https://doi.org/10.1002/ajmg.a.33405

60. Merks JHM, Caron HN, Hennekam RCM (2005) High incidence of malformation syndromes in a series of 1,073 children with cancer. Am J Med Genet A 134A:132–143. https://doi.org/10.1002/ajmg.a.30603

61. Roma D, Palma P, Capolino R, et al (2015) Spinal ependymoma in a patient with Kabuki syndrome: a case report. BMC Med Genet 16:80. https://doi.org/10.1186/s12881-015-0228-4

62. Timothy LD, Lehrke HD, Chandan VS, et al (2019) Diffuse Adenomatosis and Hepatocellular Carcinoma Treated with Liver Transplantation in an Adolescent Female with Kabuki Syndrome with a Novel KMT2D Gene Mutation. Case Rep Pediatr 2019:7983824

63. Murakami H, Tsurusaki Y, Enomoto K, et al (2020) Update of the genotype and phenotype of KMT2D and KDM6A by genetic screening of 100 patients with clinically suspected Kabuki syndrome. Am J Med Genet A. https://doi.org/10.1002/ajmg.a.61793

64. Faundes V, Goh S, Akilapa R, et al (2021) Clinical delineation, sex differences, and genotype-phenotype correlation in pathogenic KDM6A variants causing X-linked Kabuki syndrome type 2. Genet Med. https://doi.org/10.1038/s41436-021-01119-8
